# Dual transcriptional and post-transcriptional regulation by the bHLH proteins Rtg1 and RtgX in *Komagataella phaffii*

**DOI:** 10.1093/nar/gkaf1475

**Published:** 2026-01-08

**Authors:** Neetu Rajak, Richa Shah, Yash Sharma, Pundi Rangarajan

**Affiliations:** Department of Biochemistry, Indian Institute of Science, Bangalore560012, India; Department of Biochemistry, Indian Institute of Science, Bangalore560012, India; Department of Biochemistry, Indian Institute of Science, Bangalore560012, India; Department of Biochemistry, Indian Institute of Science, Bangalore560012, India

## Abstract

In *Saccharomyces cerevisiae*, the basic helix–loop–helix (bHLH) transcription factors Rtg1 and Rtg3 mediate retrograde signaling. In *Candida albicans*, the Rtg1–Rtg3 complex regulates galactose and sphingolipid metabolism and contributes to virulence. In the methylotrophic yeast *Komagataella phaffii* (formerly *Pichia pastoris*), Rtg1 (KpRtg1) controls glutamate and methanol metabolism by regulating the synthesis of GDH2 (glutamate dehydrogenase 2), PEPCK (phosphoenolpyruvate carboxykinase), and AOX1 (alcohol oxidase 1). The function of the putative Rtg3 ortholog, designated here as KpRtgX, has remained unknown. In this study, we identify KpRtgX as the missing regulatory partner of KpRtg1. Deletion of either *KpRTG1* or *KpRTGX* produces identical phenotypes, suggesting functional overlap. KpRtgX localizes to both cytosol and nucleus, activates *AOX1* transcription in the nucleus, and regulates *GDH2* and *PEPCK* post-transcriptionally in the cytosol. Notably, KpRtgX protein but not mRNA is strongly reduced in *ΔKprtg1* cells, indicating that KpRtg1 stabilizes KpRtgX. The two proteins interact through their bHLH domains when expressed in *K. phaffii* but not in *Escherichia coli*. Mutation of conserved arginine residues in the KpRtgX bHLH domain reduces its expression, implicating these residues in complex formation with KpRtg1. KpRtg1–KpRtgX complex is required both for GDH2 protein stability and for 5′ UTR–dependent translation of *PEPCK* mRNA during glutamate utilization.

## Graphical Abstract

**Graphical Abstract:**
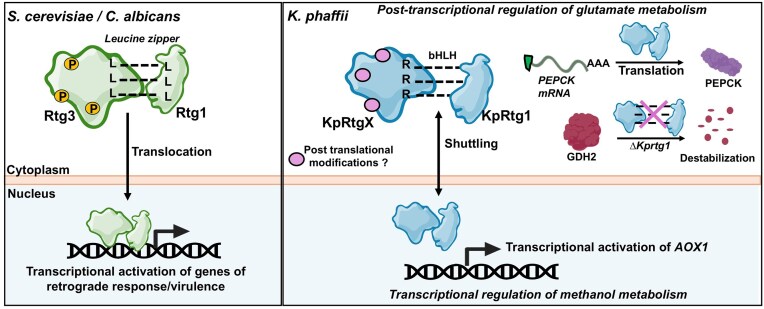

## Introduction

*Komagataella phaffii* is a methylotrophic yeast that has emerged as a robust eukaryotic host for heterologous protein production, owing to its ability to utilize a wide range of carbon sources, including glucose, glycerol, methanol, acetate, oleate, and amino acids [1–7]. The development of expression platforms in *K. phaffii* has been facilitated by extensive characterization of its metabolic pathways and the regulatory networks controlling them. Among these, the methanol-inducible system based on the promoter of the alcohol oxidase 1 (*AOX1*) gene encoding the first enzyme in the methanol utilization (MUT) pathway remains the most widely employed [8–11]. *AOX1* transcription is regulated by several zinc finger transcription factors, including Mxr1, Rop1, Trm1, Mit1, Nrg1, Mig1, and Mig2 [11–12]. In-depth mechanistic insights into these regulators have paved the way for the development of methanol-free expression systems that utilize alternative carbon sources such as glycerol [13, 14, 15]. Unlike *Saccharomyces cerevisiae, K. phaffii* can utilize glutamate as its sole carbon source, leading to the induction of enzymes essential for its metabolism, including glutamate dehydrogenase 2 (*GDH2*) and phosphoenolpyruvate carboxykinase (*PEPCK*) [16, 17–19]. This insight was leveraged to develop a glutamate-inducible expression system based on the promoter of *PEPCK* [20].

In *S. cerevisiae*, basic helix–loop–helix leucine zipper (bHLH-LZ) transcription factors Rtg1 and Rtg3 (ScRtg1–ScRtg3) form a heterodimeric complex that mediates the retrograde response, a mitochondrial-to-nuclear signaling pathway regulating genes involved in anaplerotic metabolism and mitochondrial function [21–23]. In *Candida albicans*, Rtg1 and Rtg3 (CaRtg1–CaRtg3) regulate galactose and sphingolipid metabolism and contribute to virulence, reflecting a broader functional role beyond mitochondrial stress signaling [24–29]. Engulfment of the fungal pathogen by mouse/human neutrophils triggers the production of reactive oxygen species that induces nuclear translocation of Rtg1–Rtg3 heterodimer (CaRtg1–CaRtg3) and activation of genes involved in fungal virulence. Deletion of either *CaRtg1* or *CaRtg3* or both results in reduced virulence and enhanced survival of the host [25]. In *K. phaffii*, Rtg1 (KpRtg1; NCBI accession # XP_002490029.1) localizes to cytosol and regulates the synthesis of GDH2 and PEPCK by a post-transcriptional mechanism [17–19]. Beyond glutamate metabolism, KpRtg1 also regulates *AOX1* transcription during methanol metabolism [17], suggesting a broader role in carbon metabolism regulation. Although a putative Rtg3 homolog (KpRtg3; NCBI accession # AOA70166.1) has been identified in *K. phaffii*, it does not form a heterodimer with either ScRtg1 or KpRtg1 when expressed in *Escherichia coli*. The functional role of KpRtg3 remains undefined [17]. Based on these observations, the non-canonical KpRtg3 was renamed as KpRtgX [17], hypothesizing that it may represent a functionally distinct regulator that compensates for the absence of canonical Rtg3-mediated regulation.

Here, we characterize the role of KpRtgX and its functional relationship with KpRtg1 in coordinating transcriptional and post-transcriptional regulation during methanol and glutamate metabolism, respectively. We demonstrate that *ΔKprtgX* strain exhibits a phenotype strikingly similar to *ΔKprtg1*, suggesting functional overlap. Although KpRtgX predominantly localizes in the nucleus, it has a functional role in cytosol as well during glutamate metabolism. KpRtg1 and KpRtgX regulate the same set of metabolic enzymes—GDH2 and PEPCK post-transcriptionally during glutamate metabolism and *AOX1* transcriptionally during methanol metabolism. *In vitro* assays reveal that KpRtg1 and KpRtgX expressed in *K. phaffii* but not in *E. coli* form a heterodimer. The leucine zipper in KpRtg1 is dispensable for its function and is not involved in the heterodimerization; instead, KpRtg1 and KpRtgX interact via the bHLH domain. Together, our findings establish KpRtgX and KpRtg1 as multifunctional regulators that integrate transcriptional and post-transcriptional responses to carbon-source availability in *K. phaffii*. Notably, such dual-mode regulation by homologous bHLH proteins across two distinct metabolic pathways has not previously been described for any Rtg1/Rtg3 family member in yeasts. We also identify a new cytosolic function for the Rtg1–RtgX heterodimer in regulating translation of the *PEPCK* transcript via 5′ UTR, a feature not reported in other yeasts.

## Materials and methods

### Media and culture conditions

A single colony of yeast cells was inoculated from agar (2%) plates containing YPD (1.0% yeast extract, 2.0% peptone, 2.0% glucose) into YPD liquid medium and grown overnight at 30°C in an orbital shaker at 180 rpm. Cells were washed with sterile distilled water, at least twice, and shifted to different minimal media containing 0.17% yeast nitrogen base (YNB) without amino acids and with 0.5% ammonium sulphate supplemented with 2.0% glucose (YNBD), 2.0% glycerol (YNBG), 1.0% ethanol (YNBE), 2.0% acetate (YNBA), 2% methanol (YNBM), and 1.0% glutamate (YNB Glu+). Solid media contains 2.0% agar. *Komagataella phaffii* GS115 (*his-*) was cultured in minimal media supplemented with histidine (20 µg/ml).

### Yeast and bacterial strains

Yeast strains used in this study are listed in Table 1. Yeast cells were transformed by electroporation (Gene Pulser, Bio-Rad, CA). *Escherichia coli DH5α* strain was used for cloning of recombinant plasmids and transformation was done by CaCl_2_ method.

**Table 1.** List of *K. phaffii* strains used in this study

Yeast strain	Description	Source
*GS115*	*his4*	Ref [8]
*ΔKprtg1*	*GS115, rtg1Δ::Zeo^r^*	Ref [17]
*KpRtg1*	*ΔKprtg1, his4^+^ :: (P_KPRTG1_KpRtg1-GFP)*	Ref [17]
*KpRtg1LZ*	*ΔKprtg1, his4^+^ :: (P_KPRTG1_KpRtg1^L206A,L213A^-HIS)*	This study
*KpRtg1ΔC*	*ΔKprtg1, his4^+^ :: (P_KPRTG1_KpRtg1^1-205^-GFP)*	This study
*KpRtg1ΔN*	*ΔKprtg1, his4^+^ :: (P_KPRTG1_KpRtg1^134-246^-GFP)*	This study
*KpRtg1ΔbHLH*	*ΔKprtg1, his4^+^ :: (P_KPRTG1_KpRtg1^∆131–196^-GFP)*	This study
*ΔKprtgX*	*GS115, rtgXΔ::Zeo^r^*	This study
*KpRtgX*	*ΔKprtgX, his4^+^ :: (P_KPRTGX_KpRtgX–GFP)*	This study
*KpRtgXK1^M^*	*ΔKprtgX, his4^+^ :: (P_KPRTG1_KpRtgX^K264,266,268A^-GFP)*	This study
*KpRtgXK2^M^*	*ΔKprtgX, his4^+^ :: (P_KPRTG1_KpRtgX^K287,290,293A^-GFP)*	This study
*KpRtgXNES*	*ΔKprtgX, his4^+^ :: (P_KPRTG1_KpRtgX-NES-GFP)*	This study
*ΔKprtg1-LACZ*	*GS115, rtg1Δ::Zeo^r^ : (P_AOX1_LACZ)*	Ref [17]
*ΔKprtgX-LACZ*	*GS115, rtgXΔ::Zeo^r^ : (P_AOX1_LACZ)*	This study
*KpRtgXNES-LACZ*	*ΔKprtgX, his4^+^ ::(P_KPRTG1_KpRtgX-NES-GFP)(P_AOX1_ -LacZ)*	This study
*KpRtg1^mCherry^-KpRtgX^GFP^*	*GS115, his4^+^:(P_KPRTG1_KpRtg1^mCherry^), (P_KPRTGX_KpRtgX^GFP^)*	This study
*KpRtg1^mCherry^*	*GS115, his4^+^:(P_KPRTG1_KpRtg1^mCherry^)*	This study
*KpRtg1^GST^*	*GS115, his4^+^:(P_KPAOX1_KpRtg1^GST^)*	This study
*KpRtgXR1^M^*	*ΔKprtgX, his4^+^ :: (P_KPRTG1_KpRtgX^R246,247,248,249A^-GFP)*	This study
*KpRtgXR2^M^*	*ΔKprtgX, his4^+^ :: (P_KPRTG1_KpRtgX^R257,258,259,260A^-GFP)*	This study
*ΔKprtgXKpRtg1*	*ΔKprtgX, his4^+^ :: (P_KPRTG1_KpRtg1-GFP)*	This study
*GS-KpRtg1*	*GS115, his4^+^ :: (P_KPRTG1_KpRtg1-GFP)*	This study
*ΔKprtg1KpRtgX*	*ΔKprtg1, his4^+^ :: (P_KPRTG1_KpRtgX–GFP)*	This study
*GS-P_PEPCK_PEPCK^Myc^*	*GS115, his4^+^ :: (P_PEPCK_PEPCK-MYC)*	Ref [17]
*ΔKprtg1P_PEPCK_PEPCK^Myc^*	*∆Kprtg1, his4^+^ :: (P_PEPCK_PEPCK-MYC)*	Ref [17]
*GS-P_GAPDH_PEPCK^Myc^*	*GS115, his4^+^ :: (P_GAPDH_PEPCK-MYC)*	Ref [17]
*ΔKprtg1P_GAPDH_PEPCK^Myc^*	*∆Kprtg1, his4^+^ :: (P_GAPDH_PEPCK-MYC)*	Ref [17]
*GS-P_GDH2_GDH2^His^*	*GS115, his4^+^ :: (P_GDH2_GDH2-HIS)*	Ref [17]
*ΔKprtg1P_GDH2_GDH2^His^*	*∆Kprtg1, his4^+^ :: (P_GDH2_GDH2-HIS)*	Ref [17]
*GS-P_GAPDH_GDH2^His^*	*GS115, his4^+^ :: (P_GAPDH_GDH2-HIS)*	Ref [17]
*ΔKprtg1P_GAPDH_GDH2^His^*	*∆Kprtg1, his4^+^ :: (P_GAPDH_GDH2-HIS)*	Ref [17]
GS-P_GAPDH*_PEPCK^Myc^	GS115, his4^+^ :: (P_GAPDH*_PEPCK-MYC)	This study
ΔKprtg1P_GAPDH*_PEPCK^Myc^	∆Kprtg1, his4^+^ :: (P_GAPDH*_PEPCK-MYC)	This study
*Δpepck*	*GS115, KppepckΔ :: Zeo^r^*	Ref [19]
*Δgdh2*	*GS115, Kpgdh2Δ :: Zeo^r^*	Ref [19]

### Growth kinetics and spot assay

For growth kinetics, overnight YPD-grown cells were transferred to different media with an initial A_600_ of ∼0.1 per ml of media. In the case of YNBM, methanol was replenished every 24 h. Growth was then measured as A_600_ at specified time points. For spot assay, overnight YPD-grown cells were washed and resuspended in sterile distilled water to an A_600_ of 1 per ml. 1:10 serial dilutions were made till an A_600_ of 10^−4^ per ml. Two microliters from each dilution was then spotted on solid medium.

### Reagents

Oligonucleotides were purchased from Sigma–Aldrich (Bangalore, India). Mouse anti-Myc tag antibody was purchased from Merck Millipore (Bangalore, India). Mouse anti-GFP and anti-GST were purchased from Santa Cruz Biotechnology Inc. (Santa Cruz, CA), and mouse anti-His tag antibodies were purchased from Cell Technology (Denvers, MA). Anti phosphoglyerate kinase (PGK) antibody was generated by immunizing rabbit with purified *K. phaffii* PGK protein. Restriction enzymes and T4 DNA ligase were purchased from New England Biolabs (Ipswich, MA). DNA polymerases were purchased from GeNei (Bangalore, India) and Thermo Fisher Scientific (Waltham, MA).

### Total RNA isolation, reverse transcription-PCR, and quantitative PCR

Total RNA was isolated using an RNA isolation kit (Promega, WI). Complementary DNA (cDNA) was prepared using 1 µg of DNase-treated RNA followed by quantitative PCR (q-PCR) using the StepOnePlus Real-Time PCR system (Thermo Fisher Scientific). Levels of mRNA expression in the deletion strain relative to the wild type were normalized to the internal control i.e. 18s rRNA transcript levels. The comparative ΔΔCt method was used for data analysis and further interpretation. Sequence of primers used for qPCR is listed in Supplementary material and Supplementary Table S1.

### Live cell imaging

Cells were initially grown overnight in YPD medium, then transferred to the appropriate media. After 6 h of induction, 500 μl of culture was harvested by centrifugation at 5000 RPM for 2 min. The cell pellet was washed twice with 1× phosphate buffered saline (PBS) (137 mM NaCl, 2.7 mM KCl, 4.3 mM Na₂HPO_4_, and 1.47 mM KH₂PO_4_), adjusted to pH 7.4, and finally resuspended in 500 μl of 1× PBS. Hoechst 33342 (10 μl) was added to the resuspended cells, which were then incubated at 30°C for 10 min with shaking at 180 RPM. Following staining, cells were washed twice again with 1× PBS. Fluorescence images were acquired using an Olympus IX83 widefield microscope equipped with a CoolLED PE-4000 LED light source and a Prime-BSI sCMOS camera. Imaging was performed using a 100× oil-immersion objective with a 1 μm step size across three z-slices. Raw images were processed using Olympus CellSens Dimension (version 3.1) software for 3D deconvolution, and maximum-intensity projection images were used for final representation.

### Yeast cell lysate preparation using glass bead lysis and western blotting

Yeast cells grown in YNBGlu/YNBM were centrifuged at 7000× *g* for 3 min to pellet down cells, which are then dissolved in lysis buffer (20 mM Tris–HCl, pH 8.0, 400 mM NaCl, 10 mM MgCl_2_, 7 mM β-mercaptoethanol, 10% glycerol, 1× protease inhibitor cocktail). Cells were lysed at 4°C using a bead beater or vortexing in the presence of glass beads. Protein concentration in the lysate was quantified using Bradford reagent. Approximately 50–100 µg of cell lysates were subjected to sodium dodecyl sulphate-polyacrylamide gel electrophoresis (SDS–PAGE) followed by western blot analysis. Proteins were detected using primary antibodies (anti-GFP antibody, Abcam) at dilutions suggested by the manufacturer and HRP-conjugated secondary antibodies. PGK antibodies which were obtained by immunizing rabbits with recombinant *K. phaffii* PGK protein, were used as primary antibodies, followed by HRP-conjugated anti-rabbit secondary antibodies, for checking loading control. Bands were visualized by chemiluminescence substrate (G-Biosciences, St. Louis, USA) according to the manufacturer’s protocol.

### Silver staining

Silver staining of proteins resolved on SDS polyacrylamide gels was performed following a previously reported protocol [18]. After electrophoresis, gels were fixed in a solution (solution A) containing 50% methanol, 10% acetic acid, and 40% Milli-Q water for at least 30 min or overnight. Fixed gels were washed three times with 50% ethanol for 20 min each to remove residual fixatives. For sensitization, gels were incubated in 0.02% (*w/v*) sodium thiosulfate solution for 1 min. The gels were then stained with 0.1% (*w/v*) silver nitrate for 20 min at room temperature. After optional rinsing with water, bands were developed using a freshly prepared solution of 3% (*w/v*) sodium carbonate containing 0.05% (*v/v*) formaldehyde. The development process was monitored visually, and once desired band intensity was achieved (typically within 2–10 min), the reaction was stopped by immersing the gel in solution A for 10 min. Gels were then rinsed with water and either imaged directly or stored in water for further analysis.

### GST pull-down assay

*Escherichia coli* cells (*BL21DE3*) expressing GST-tagged anti-GFP nanobody (Addgene plasmid #61838, [30]) were suspended in 1× PBS (137 mM NaCl, 2.7 mM KCl, 100 mM Na_2_HPO_4_, and 2 mM KH_2_PO_4_, pH 7.4) containing 1 mM PMSF, 1 mM EDTA, 1 mM DTT, 10 mg/ml lysozyme, and 1% Triton X-100 and kept on ice for 20 min. Cells were then sonicated, and cell lysates containing GST-tagged anti-GFP nanobodies were incubated with glutathione resin (G-Biosciences, St. Louis, USA) at 4°C for 1 h. Nanobody-bound glutathione resins were harvested by brief centrifugation followed by washes with 1× PBS containing 1 mM PMSF. GST-tagged anti-GFP nanobody-bound glutathione resins were incubated with yeast (*GS115*) whole-cell protein lysate containing GFP-expressing *P_AOX1_, P_GDH2_*, or *P_PEPCK_* at 4°C for 2–3 h. Post-interaction, resins were centrifuged briefly and washed at least thrice with cold 1× PBS. Bound proteins were resolved by SDS‒PAGE and visualized by Coomassie Brilliant Blue R-250 staining.

### Protein–protein interaction studies

For Rtg proteins expressed in *E. coli*, the pull-down assays were performed as previously reported [17]. For protein–protein interaction of RtgX-GFP and Rtg1-mCherry expressed in *K. phaffii*, anti-GFP nanobody bound to GST-beads were purified as described above in the GST pull-down assay. The GST beads carrying anti-GFP nanobodies were used as bait and incubated with lysates containing RtgX-GFP alone and lysates containing RtgX-GFP as well as Rtg1-mCherry. Around 100 μl of anti-GFP nanobody-GST beads was incubated with 2 mg total lysate on a nutator at 4°C for 2 h. After interaction, beads were washed with wash buffer A (1× PBS, 0.2% Triton X). Similarly, to detect the interaction of RtgX-GFP and Rtg1-GST, the lysate containing Rtg1-GST was incubated with GST beads on a nutator at 4°C for 2 h and washed with wash buffer A. Rtg1-GST bound to GST beads acts as a bait and is further used for interaction in the same way as anti-GFP nanobodies.

### Mapping the 5′ end of *PEPCK* mRNAs by RNA ligase-mediated rapid amplification of cDNA ends

Total RNA was isolated from *GS115* using an RNA isolation kit (RNeasy Mini Kit, Qiagen). Ten micrograms of RNA was treated with calf intestine alkaline phosphatase as per the protocol mentioned in the Ambion RLM-RACE Kit. The RNA was then treated with tobacco acid pyrophosphatase and ligated with 45 bp RNA adaptor oligonucleotide using the T4 RNA ligase provided in the kit. The reaction was then subjected to reverse transcription using the enzyme provided in the kit to produce full-length adapter-ligated cDNA. The cDNA prepared was taken as a template for nested PCR. Specific reverse primers were designed for each target, i.e. PEPCK (RP_PEPCK_RLM_1, RP_PEPCK_RLM_2) and GAPDH (RP_GAP_RLM_1, RP_GAP_RLM_2), keeping the forward primer (outer primer and inner primer) against adapter sequence the same (provided in the kit). The primers used were purchased from Sigma–Aldrich, India, and are listed in Supplementary material and Supplementary Table S1. The amplified PCR product was gel purified for sequencing to map the 5′ UTR of *GAPDH* and *PEPCK* mRNAs.

### β-Galactosidase assay

β-Galactosidase activity was measured using ortho-nitrophenyl-d-galactopyranoside as the substrate. Cells were cultured in YNBM for 12–16 h. A 50 μg sample of whole cell protein lysate was diluted with 1 ml of Z-buffer (60 mM Na₂HPO_4_, 40 mM NaH₂PO_4_, 10 mM KCl, 1 mM MgCl₂, pH 7.0) and incubated for 5 min at 30°C. Then, 0.2 ml of a 4 mg/ml ortho-nitrophenyl-d-galactopyranoside stock solution (diluted in 100 mM sodium phosphate buffer, pH 7.0) was added, and the reaction was performed at 30°C for 5–10 min. Then, 0.25 ml of 2 M Na₂CO₃ was added to stop the reaction. The release of o-nitrophenol was quantified by measuring absorbance at 420 nm. Enzyme activity was defined as the amount (nanomoles) of o-nitrophenol released per minute per microgram of protein lysate under these conditions.

### Generation of *K. phaffii* strains

The nucleotide sequence of the primers used in PCR reactions for the generation of various constructs and *K. phaffii* strains are listed in Supplementary material and Supplementary Table S2.

### Generation of *KpRtg1LZ*^*mut*^, *KpRtg1∆C, KpRtg1∆N*, and *∆KpRtg1∆bHLH* strains

To generate *KpRtg1LZ^mut^*, a fragment containing KpRtg1 with mutations—*L206A, L213A*, along with 1 kb KpRtg1 promoter—was amplified by two-step PCR using mutated primers. The first PCR was performed with primer pair A1 and A2 and *GS115* genomic DNA as a template. The first amplification generated a fragment containing *KpRtg1* 1 kb promoter and *KpRtg1* (1–654 bp). The second PCR was performed with primer pair A3 and A4 (mutated sequence in A3 is underlined), using *GS115* genomic *DNA* as a template. Both PCR fragments were purified (NucleoSpin™ Gel and PCR Clean-up) and used as a template for the final overlap extension PCR using primers A1 and A4. The generated fragment contains overlap regions with *pIB3* vector. The generated fragment was cloned into KpnI site of *pIB3* using 1× NEB HiFi Builder and transformed into *E. coli DH5α* using the heat-shock method. The positive clones were selected by plating on ampicillin-containing LB agar plates. Recombinant plasmid containing *KpRtg1LZ^mut^* was amplified, linearized with SalI, and transformed into *ΔKprtg1* by electroporation. Recombinant clones were selected by plating on YNBD His^−^ plates. Clones expressing His-tagged *KpRtg1LZ^mut^* were confirmed by pull-down with Ni-NTA beads followed by silver staining.

For generation of truncated *KpRtg1∆C* (∆205–246 amino acids) His fusion proteins, along with ∼1 kb of *KpRTG1* promoter. The cassette containing 1 kb *KpRtg1* promoter and *KpRtg1* 1–205 amino acids was amplified using *GS115* genomic DNA as a template and primers—forward primer A1 and reverse primer B1. The fragment was PCR-purified (NucleoSpin™ Gel and PCR Clean-up), and concentration was measured using Thermo-NanoDrop2000. Cloning vector *pIB3* was linearized with *Kpn1* and purified. The PCR-generated fragment was cloned into *pIB3* using 1× NEB HiFi Builder and transformed into *E. coli DH5α* using the heat-shock method. The positive clones were selected by plating on LB-ampicillin agar plates. Recombinant plasmid containing *KpRtg1ΔC* was amplified, linearized with SalI, and transformed into *ΔKprtg1* by electroporation. Recombinant clones were selected by plating on YNBD His^−^ plates. Clones expressing His-tagged *KpRtg1ΔC* were confirmed by pull-down with Ni-NTA beads followed by silver staining.

Similarly, to generate *KpRtg1∆N*, a truncated KpRtg1 (∆1–134 amino acids) GFP was amplified from template *P_KPRTG1_KpRtg1^GFP^* [17]. The fragment was generated using primer pair C1 and C2. The second fragment containing KpRTG1 ∼1 kb promoter was amplified from *GS115* genomic *DNA*, using primer pair—C3 and C4. Both the fragments contain overlapping regions with each other as well as with vector *pGHYB*. Both fragments were purified and cloned into XhoI site of *pGHYB* using NEB HiFi Builder and transformed into *E. coli DH5α* using the heat-shock method. The positive clones were selected by plating on LB hygromycin agar plates. Recombinant plasmid containing *KpRtg1ΔN* was amplified, linearized with AvrII, and transformed into *ΔKprtg1* by electroporation. Recombinant clones were selected by plating on YPD Hyg plates. Clones expressing GFP-tagged *KpRtg1ΔN* were confirmed by western blotting using anti-GFP antibody.

To generate the *KpRtg1∆bHLH* strain, a fragment of *KpRtg1* (1–396 bp) along with ∼1 kb KpRtg1 promoter (*P_KpRTG1_*) was amplified from vector *P_KpRTG1_KpRtg1^GFP^* generated in [17]. For amplification, the primer pair used was D1 and D2. The second fragment containing *KpRtg1* (591 to 741 bp) and *GFP* was amplified from *P_KpRTG1_KpRtg1^GFP^* using primer pair D3 and D4. Both the PCR products were purified and used as a template for the final overlap extension PCR using primer pair D1 and D4. The final product was purified and digested with XhoI and HindIII and ligated into *pGAPBA* (linearized with XhoI and HindIII), followed by transformation into *E. coli DH5α* competent cells. Recombinant plasmid containing *P_KpRtg1_-KpRtg1∆bHLH* was amplified, linearized with AvrII, and transformed into *ΔKprtg1* by electroporation. Recombinant clones were selected by plating on Blasticidin-containing YPD plates. Clones expressing *KpRtg1∆bHLH* were confirmed by western blotting using anti-GFP antibody.

### Generation of *ΔKprtgx, KpRtgX, KpRtgX-NES, KpRtgX-K1*^*M*^, and *KpRtgX-K2*^*M*^ strains and *KpPro*_*KpRtgX–GFP*_ vectors

To generate a *ΔKprtgX* strain, the *KpRtgX* coding sequence was replaced with a Zeocin resistance (Zeocin^R^) expression cassette. First, ∼1 kb of the RtgX promoter was amplified using the primer pair E1 and E2. The Zeocin^R^ cassette (∼1.2 kb) was amplified from the *pGAPZA* vector (Invitrogen) using primer: forward primer E3 and reverse primer E4. Separately, ∼1 kb of the *KpRtgX* terminator region was amplified from *GS115 genomic DNA* using primers E5 and E6. All three PCR products were gel-purified and used as templates (50 ng each) for a final overlap-extension PCR using the forward primer from the promoter and the reverse primer from the terminator E1 and E6. The resulting fusion PCR product was purified and transformed into *GS115* cells. Transformants were selected on zeocin-containing plates, and gene deletion (*ΔKprtgX*) was confirmed by PCR using the primer pair E7 and E8.

To express *GFP-tagged KpRtgX* in *K. phaffii*, the gene encoding *KpRtgX* along with ∼1 kb of its native promoter was amplified from *GS115* genomic DNA using primers F1 and F2. *GFP* was amplified from the *pREP41GFP* vector using primers F3 and F4. The KpnⅠ site was introduced in the forward primer F1 of the *KpRtgX* promoter and the NotⅠ site in the reverse primer F4 of GFP, allowing directional cloning. After purification, these PCR products were used as templates in a final PCR using primers containing KpnⅠ and NotⅠ sites. The final product (*P_KPRTGX_KpRtgX–GFP*) was purified, digested with KpnⅠ and NotⅠ, and cloned into the *pGHYB* vector. The recombinant plasmid was linearized with AvrⅡ and transformed into the *ΔKprtgX* strain. Hygromycin-resistant transformants were selected and screened via western blotting using an anti-GFP antibody to confirm expression.

To generate the *KpRtgX-NES* fusion construct, a two-step PCR strategy was used. The first PCR product consisted of the *KpRtgX* coding region along with its native ∼1 kb promoter, amplified from *GS115* genomic *DNA* similar to as mentioned above. The second PCR product contained *GFP* fused to a C-terminal NES (nuclear export signal). This fragment was amplified from the *pREP41GFP* vector using the primer pair G1 and G2. The NotⅠ site was incorporated into the reverse primer for directional cloning, and the two PCR products were gel-purified and used as templates for a final fusion PCR using primers F1 and G2 containing KpnⅠ and NotⅠ restriction sites. The resulting full-length *P_KPRTGX_-KpRtgX-NES* construct was digested with KpnⅠ and NotⅠ and ligated into the *pGAPBA* vector digested with the same enzymes. Recombinant plasmids were amplified in *E. coli DH5α*, verified by sequencing, and linearized with AvrⅡ prior to transformation into the *ΔKprtgX* strain. Transformants were selected on blasticidin-containing agar plates, and positive clones were confirmed by Western blotting using an anti-GFP antibody (Abcam).

To generate *KpRtgXK1^M^* and *KpRtgXK2^M^* fusion constructs under the control of the native *KpRtgX* promoter, a modular expression vector was first created with the *KpRtgX* promoter–MCS–GFP arrangement. First, the *KpRtgX* promoter (∼1 kb) was PCR-amplified from *GS115* genomic *DNA* using the primer pair H1 and H2. This fragment was purified and cloned into the KpnⅠ site of the *pGHYB* vector using NEBuilder HiFi DNA Assembly Master Mix (NEB), resulting in a *pGHYB* construct containing the *KpRtgX* promoter upstream of a multiple cloning site (MCS). Next, the GFP coding sequence, lacking an ATG start codon, was PCR-amplified from the *pREP41GFP* vector using primers H3 and H4. The amplified GFP fragment was gel-purified and inserted at the *NotⅠ* site downstream of the MCS using HiFi assembly, yielding the parent expression vector containing the *KpRtgX* promoter–MCS (with BamHI and XhoI sites)–GFP. This vector is called *KpPro_KpRtgX–GFP_*.

Synthetic genes K1 and K2 (Supplementary material and Supplementary Table S3), each containing BamHI and XhoI restriction sites and overlapping regions with XhoI flanking sequence, were procured from Twist Bioscience. These fragments were cloned into the Xho1 site between the *KpRtgX* promoter and GFP using NEB HiFi builder, thereby creating *KpRtgXK1^M^* and *KpRtgXK2^M^* constructs. The resulting recombinant plasmids were propagated in *E. coli* DH5α, verified by sequencing, and linearized with AvrⅡ. These were then transformed into *ΔKprtgX*. Transformants were selected on YPD plates containing hygromycin, and positive colonies were screened by fluorescence microscopy for GFP expression.

### Generation of *K. phaffii, KpRtg1–mCherry, KpRtgX–GFP, KpRtg1–mCherry*, and *KpRtg1–GST* strains

To generate *KpRtg1–mCherry*, a cassette containing the *KpRtg1* promoter with *KpRtg1* coding region was amplified from *GS115* gDNA using primer pair: I 1 and I 2. The generated *P_KpRTG1_-KpRtg1* fragment contains overlapping regions with the *pIB3-mCherry* vector (made in lab, unpublished data). The fragment was purified and cloned into the XhoI site of *pIB3-mCherry*, followed by transformation into *E. coli* competent cells. Positive clones were screened by plating on ampicillin-containing LB agar plates. Recombinant plasmid was linearized using *SalI* and transformed into *GS115* and *∆Kprtg1*. Positive clones were screened by performing fluorescence microscopy and analysing mCherry signal.

To express GST–KpRtg1 in *K. phaffii*, a cassette containing GST–KpRtg1 was amplified from *pGEX* vector containing *GST-Rtg1* [17] using primer pair: J1 and J2. The generated *GST–KpRtg1* fragment contains overlapping regions with the *pPICZc* vector (gift from Dr Sarvanan Palani). The fragment was purified and cloned into the EcoR1 and KpnI sites of *pPICZc*. Positive clones were screened by plating on zeocin-containing LB agar plates. Recombinant plasmid was linearized using *PmeI* and transformed into *GS115*. Ten colonies from the transformed plate were grown in YPD, pelleted, and transferred to BMMY for *P_AOX1_* induction. From this culture, around 1 ml of culture media was pelleted down and lysed. Positive clones were screened by performing western blotting using an anti-GST (Santa Cruz) antibody to confirm expression.

To generate *KpRtg1–mCherry, KpRtgX–GFP, KpRtgX–GFP* generated in the previous section, was linearized with AvrII and transformed into *GS*-*KpRtg1–mCherry* strain by electroporation. Positive clones were screened by performing fluorescence microscopy and analysing GFP signals.

### Generation of *KpRtgX-R1*^*M*^, *KpRtgX-R2*^*M*^, *ΔKprtgxKpRtg1*, and *ΔKprtg1KpRtgX* strains

To generate the *KpRtgX-R1^M^* (R246A, R247A, R248A, R249A) and *KpRtgX-R2^M^* (R257A, R258A, R259A, R260A), fused with *GFP*, a two-step PCR strategy was used for each. To introduce the R1 mutations using site-directed mutagenesis, a fragment of *KpRtgX* was amplified from *GS115* genomic DNA using primer pair—K1 and K2. Similarly, second fragment of *KpRtgX* was amplified from *GS115* gDNA using primer pair—K3 and K4. Overlap extension PCR was performed to fuse both fragments. For that, the primer pair K1 and K4 was used with both the fragments taken as a template. The generated *KpRtgXR1^M^* also contains overlapping regions to assist its integration into the XhoI site of the previously generated vector *KpPro_KpRtgX–GFP_* using NEB HiFi Builder, creating *KpRtgXR1^M^* constructs.

Similarly, to introduce the R2 mutations (R257A, R258A, R259A, R260A) using site-directed mutagenesis, a fragment of *KpRtgX* was amplified from *GS115* genomic DNA using primer pair—K1 and L1. Similarly, second fragment of *KpRtgX* was amplified from *GS115* genomic *DNA* using primer pair—L2 and K4. Overlap extension PCR was performed to fuse both fragments. For that, primer pair K1 and K4 was used, with both the fragments taken as a template. The generated *KpRtgXR2^M^* also contains overlapping regions to assist its integration into the XhoI site of the previously generated vector *KpPro_KpRtgX–GFP_* using NEB HiFi Builder, creating *KpRtgXR2^M^* constructs.

The resulting constructs—*KpRtgXR1^M^* and *KpRtgXR2^M^—*were transformed into *E. coli* competent cells, verified by sequencing, and linearized with AvrⅡ. These were then transformed into *ΔKprtgX* by electroporation. Transformants were selected on YPD plates containing hygromycin, and positive colonies were screened by analyzing GFP expression using fluorescence microscopy.

To generate *ΔKprtg1KpRtgX*, the construct, *P_KPRTGX_KpRtgX–GFP*, generated in the previous sections, was linearized with AvrII and was transformed into *ΔKprtg1* by electroporation. Transformants were selected on YPD plates containing hygromycin, and positive colonies were screened by fluorescence microscopy for GFP expression.

To generate *ΔKprtgXKpRtg1* and *GSKpRtg1*, the construct, *P_KPRTG1_KpRtg1-GFP*, generated in [17], was linearized with AvrII and was transformed into *ΔKprtgX* and *GS115* by electroporation. Transformants were selected on YPD plates containing hygromycin, and positive colonies were screened by analyzing GFP expression using fluorescence microscopy.

### Generation of *GS-P*_GAPDH_*PEPCK^*Myc*^ and *ΔKprtg1P*_GAPDH_*PEPCK^*Myc*^ strains

To generate a cassette containing *GAPDH* promoter region (−1048 to −65 bp) fused with *PEPCK* coding region starting from −30 bp (TSS), two separate fragments were amplified. The first fragment containing GAPDH promoter (−1048 to −65) with overlapping regions of *pIB3* vector and *PEPCK* was amplified using primer pair—M1 and M2. The second fragment containing *PEPCK* sequence starting from −30 bp with MYC tag at the 3′ end, flanked with overlapping regions of first fragment and *pIB3*, was amplified using primer pair—M3 and M4. Both the fragments were cloned into *pIB3* at the Kpn1 site using NEB HiFi builder, followed by transformation into *E. coli* competent cells. The positive clones were selected by plating on LB Amp agar plates. Recombinant plasmid containing P_GAPDH*_PEPCK-MYC was amplified, linearized with SalI, and transformed into *GS115* and *ΔKprtg1* by electroporation. Recombinant clones were selected by plating on YNBD His^−^ plates. Positive clones were confirmed by Western blotting using an anti-myc antibody (Santa Cruz).

### Statistical analysis

Unpaired Student’s *t*-test and one way ANOVA was carried out using GraphPad Prism 5 software. Error bar denotes mean ± S.D. (biological replicates, *n* ≥ 3). *P*-value is mentioned on the bar of each figure: *P < .05; **P < .005; ***P < .0005, and n.s., not significant.

## Results

### KpRtgX localizes to both nucleus and cytoplasm and is required for growth on non-fermentable carbon sources

**Figure 1.**
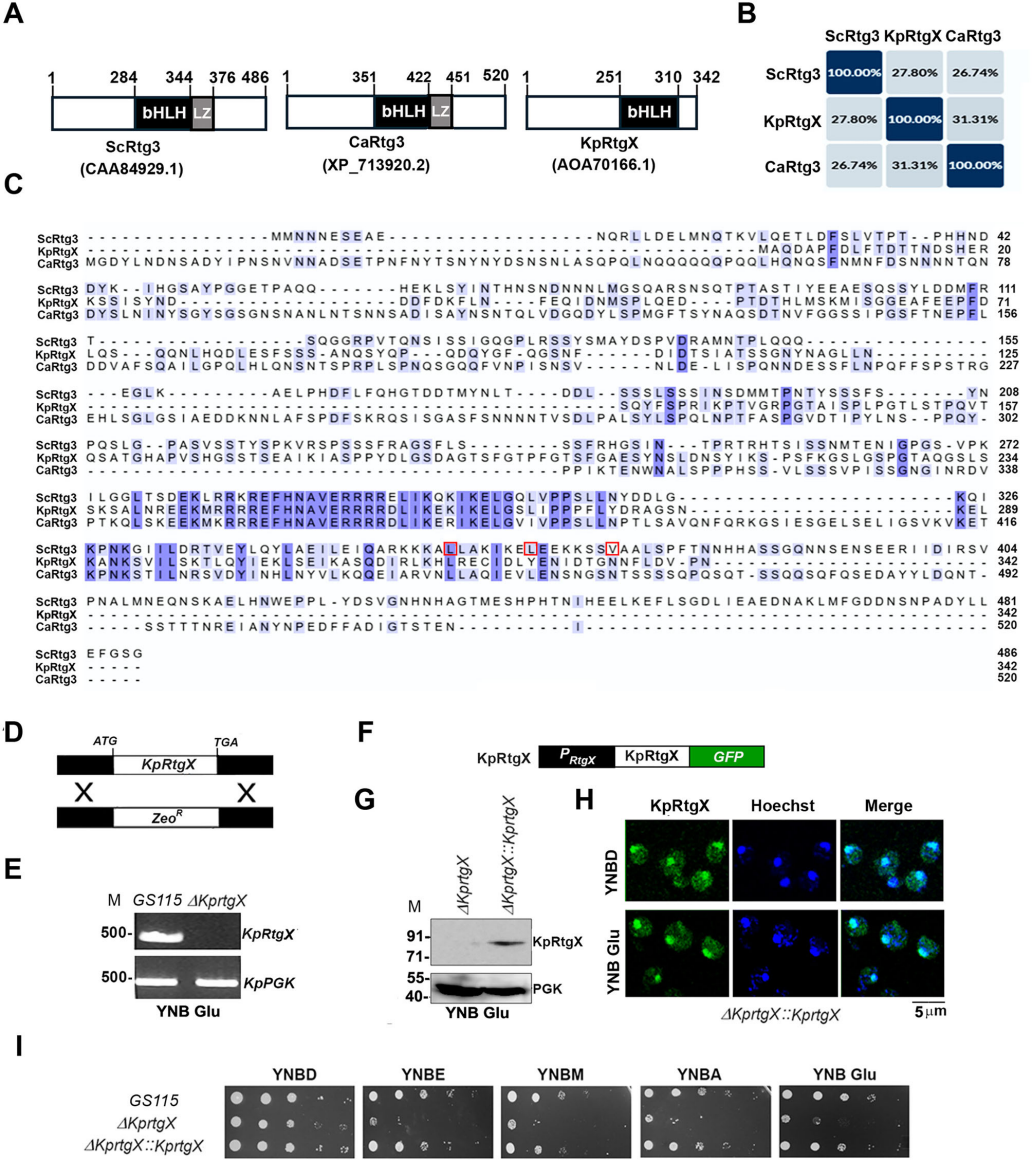
Comparison of Rtg3/RtgX of *S. cerevisiae, C. albicans*, and *K. phaffii* and functional characterization of KpRtgX. (**A**) Schematic representation of ScRtg3, CaRtg3, and KpRtg3. NCBI accession numbers are shown in parentheses. bHLH and LZ domains are indicated. Numbers indicate the position of amino acids. (**B**) Percent identity matrix comparing amino acid sequence identity among ScRtg3, CaRtg3, and KpRtg3. (**C**) Multiple sequence alignment of ScRtg3, CaRtg3, and KpRtg3. Amino acids identical in at least two proteins are shown in violet colour. Numbers indicate amino acid positions. L358 and L365, and V372, which contribute to leucine zipper formation in ScRtg3, are boxed. (**D**) Strategy for the generation of *ΔKprtgX*. RtgX coding region was replaced by a zeocin resistance (*Zeo^R^*) expression cassette. (**E**) Confirmation of *RtgX* deletion in *ΔKprtgX* by PCR amplification of genomic DNA isolated from *GS115* and *ΔKprtgX*. Cells were cultured in YNBD for 12 h. *Komagataella phaffii PGK* encoding phosphoglycerate kinase served as a control. Gene-specific primers were designed to amplify a 500 bp region from the coding region of *KpRtgX* and *KpPGK*. (M), DNA molecular weight marker (bp). (**F**) KpRtgX–GFP expression cassette. (**G**) Western blot analysis of KpRtgX using anti-GFP antibodies. PGK served as a loading control. (M), protein molecular weight markers (kDa). (**H**) Subcellular localization of KpRtgX transformed into *ΔKprtgX* as examined by fluorescence microscopy of cells cultured in YNBD and YNB Glu for 6 h. Hoechst 33342 was used to stain the nucleus. (**I**) Spot assay of different *K. phaffii* strains as shown.

*Komagataella phaffii* strains used in this study are described in Table 1. Amino acid sequence analysis reveal that KpRtgX exhibits limited sequence identity with ScRtg3 (27.8%) and CaRtg3 (31.3%) underscoring notable divergence among orthologs (Fig. 1A and B). Notably, leucine residues in ScRtg3 at positions 358 and 365, together with valine at 372, are essential for leucine zipper formation (Fig. 1C) [21–23]. Comparative analysis revealed conservation of L358 and L365 in CaRtg3, whereas only L358 was retained in KpRtgX (Fig. 1C). To investigate the function of KpRtgX in *K. phaffii*, we deleted *RTGX* to generate a *∆KprtgX* strain (Fig. 1 D and E) and a complemented strain (*∆KprtgX::KpRtgX*) expressing GFP-tagged KpRtgX (Fig. 1F). Expression of the tagged protein was confirmed by immunoblotting with anti-GFP antibodies (Fig. 1G). Fluorescence microscopy revealed that KpRtgX localized to both cytoplasm and nucleus (Fig. 1H). Growth assays demonstrated that *∆KprtgX* exhibited severe defects on ethanol (YNBE), methanol (YNBM), acetate (YNBA), and glutamate (YNB Glu), but not on glucose (YNBD). Expression of KpRtgX^GFP^ reversed the growth defect (Fig. 1I). These findings suggest that KpRtgX regulates utilization of multiple non-fermentable carbon sources.

### Lysine clusters in KpRtgX form an authentic nuclear localization signal, direct nuclear import required for methanol but dispensable for glutamate metabolism

**Figure 2.**
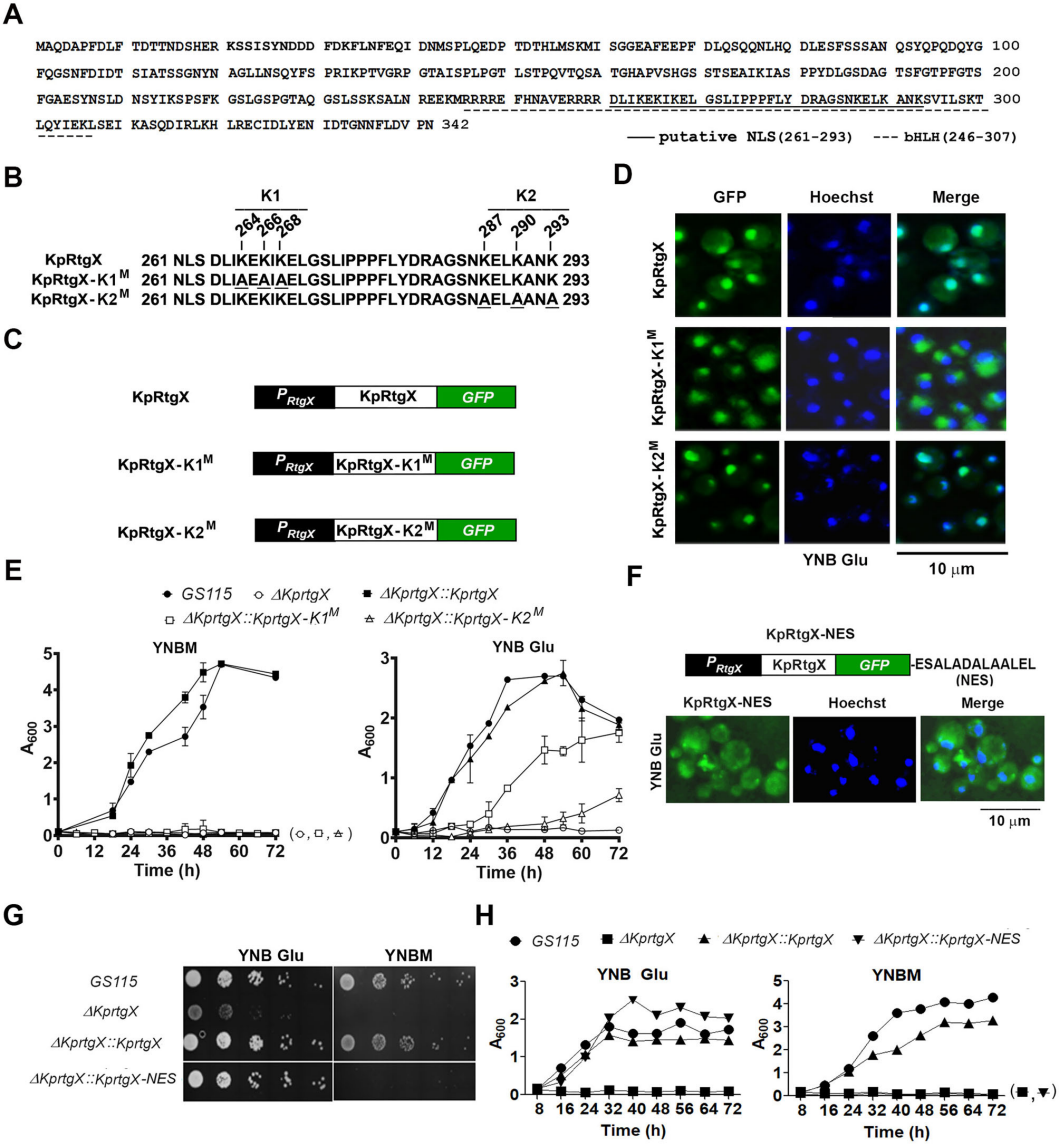
Characterization of the NLS of KpRtgX and analysis of its nuclear and cytosolic functions. (**A**) Amino acid sequence of KpRtgX. Putative NLS and bHLH domains are highlighted. (**B**) Putative bipartite NLS of KpRtg1 harbouring two clusters of lysine residues (K1 and K2), which were mutated to alanine (K1^M^ and K2^M^) as indicated. (**C**) Schematic diagram of KpRtgX, KpRtgX-K1^M^, and KpRtgX-K2^M^. (**D**) Fluorescence microscopic images of KpRtgX, KpRtgX-K1^M^, and KpRtgX-K2^M^. Hoechst 33342 was used to stain the nucleus. (**E**) Growth of different *K. phaffii* strains in YNBM and YNB Glu as indicated. Error bars indicate mean ± S.D. *n *= 3. (**F**) Generation of KpRtgX-NES construct and analysis of their subcellular localization by fluorescence microscopy. (**G, H**) Analysis of growth of different *K. phaffii* strains cultured in YNB Glu and YNBM for 72 h as indicated. Error bars indicate mean ± S.D. *n *= 3.

Amino acid sequence analysis of KpRtgX revealed a putative bipartite nuclear localization signal (NLS) spanning residues 261–293 within the bHLH domain of KpRtgX (Fig. 2A and B). This region contains two lysine-rich clusters: K264, K266, K268 (K1), and K287, K290, K293 (K2) (Fig. 2B). To probe their role, we generated two mutant constructs: KpRtgX-K1^M^ (K264A/K266A/K268A) and KpRtgX-K2^M^ (K287A/K290A/K293A) (Fig. 2B) and expressed them as GFP-fusion proteins in the *∆KprtgX* background (Fig. 2C). Fluorescence imaging revealed that KpRtgX-K1^M^ failed to accumulate in the nucleus, whereas KpRtgX-K2^M^ retained nuclear localization (Fig. 2D). Thus, the cluster of residues K264, K266, and K268 is collectively essential for the efficient nuclear localization of KpRtgX. On performing growth kinetics, we observed that KpRtgX-K1^M^, which localizes in cytoplasm, restored growth in YNB Glu but not YNBM medium (Fig. 2E). These observations indicate that nuclear localization of KpRtgX is essential for its function during methanol metabolism. On the other hand, KpRtgX has a cytoplasmic function during glutamate metabolism. Interestingly, KpRtgX-K2^M^, despite localizing to the nucleus, exhibited growth defects in YNBM as well as YNB Glu (Fig. 2D and E), suggesting that K287, K290, and K293 have important functions, independent of nuclear localization. The slow growth by KpRtgX-K1^M^ in YNB Glu despite its cytosolic localization and the loss of function of KpRtgX-K2^M^ even when localized in the nucleus suggest that the lysine residues are not solely involved in nuclear localization but may have additional roles. To confirm the compartment-specific function of KpRtgX, we fused a heterologous nuclear export signal (NES) [31–33] to its C-terminus, generating KpRtgX-NES, which localized to the cytoplasm (Fig. 2F). This approach enabled us to alter its subcellular localization without changing any amino acid residue in KpRtgX. Functional analysis showed that KpRtgX–NES fully restored growth in YNB-Glu but failed to support growth in YNBM (Fig. 2G and H). Thus, exclusion of KpRtgX from the nucleus using either an NES or mutating the K264, K266, and K268 cluster abrogates its function during methanol metabolism, confirming that nuclear localization of KpRtgX is essential for methanol metabolism but dispensable for glutamate metabolism.

### KpRtgX post-transcriptionally regulates *GDH2* and *PEPCK* during glutamate metabolism and transcriptionally activates *AOX1* during methanol metabolism

**Figure 3.**
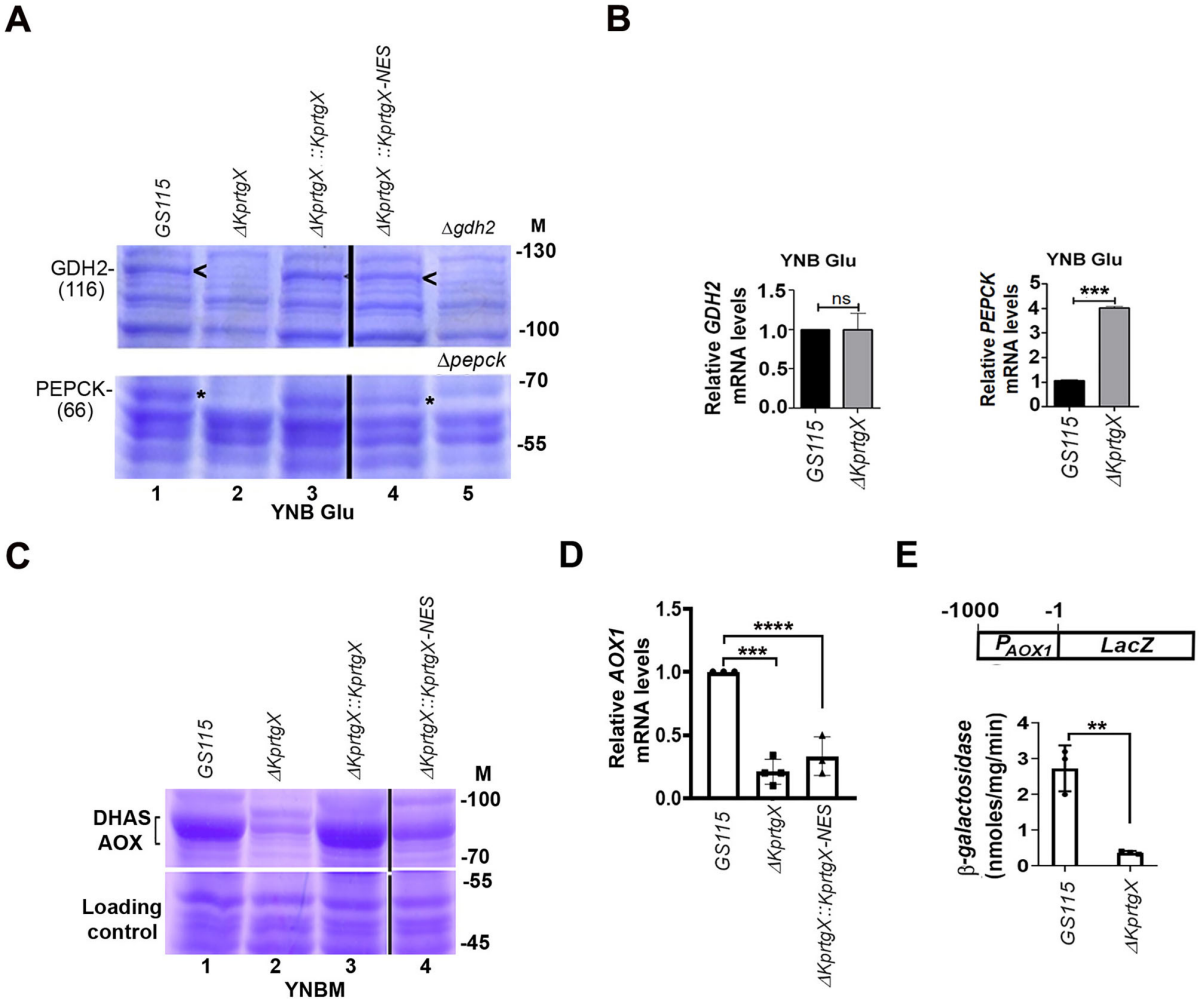
Identification of KpRtgX targets during glutamate and methanol metabolism. (**A**) SDS–PAGE of cell lysates of different *K. phaffii* strains cultured in YNB Glu for 6 h followed by Coomassie Brilliant Blue staining of the gel. GDH2 and PEPCK bands were identified by their absence in *Δgdh2* and *Δpepck* strains [20, 22], respectively. (M), protein molecular weight markers (kDa). (**B**) Quantitation of *GDH2* and *PEPCK* mRNA levels by qPCR in cells cultured in YNB Glu for 6 h. (**C**) SDS–PAGE of cell lysates of different *K. phaffii* strains cultured in YNBM for 6 h followed by Coomassie Brilliant Blue staining of the gel. (M), protein molecular weight markers (kDa). (**D**) Quantitation of *AOX1* mRNA levels by qPCR in cells cultured in YNBM for 6 h. (**E**) Schematic representation of *P_AOX1_-LacZ* expression cassette and quantitation of β-galactosidase activity in cell lysates of *GS115* and *ΔKprtgX*. In all the graphs, error bars indicate mean ± S.D. *n* = 3. *P*-value summary is mentioned on the bar of each figure. *P < .05, **P < .005, ***P < .0005. ns, not significant.

Glutamate-inducible synthesis of GDH2 and PEPCK is essential for glutamate metabolism in *K. phaffii* [17–20]. Both proteins appear as 116 kDa and 66 kDa Coomassie-stainable bands in SDS–polyacrylamide gels of lysates of wild-type *GS115* cultured on YNB Glu but disappear in *Δgdh2* and *Δpepck* strains (Fig. 3A, compare lanes 1 and 5), consistent with previous mass spectrometry confirmation of their identity [17]. We examined these proteins in *∆KprtgX*. GDH2 and PEPCK were markedly reduced in *∆KprtgX* lysates (Fig. 3A, lane 2), but their levels were restored upon expression of wild-type KpRtgX and KpRtgX-NES (Fig. 3A, lanes 3 and 4). Importantly, GDH2 and PEPCK transcript levels were not decreased in *∆KprtgX*, as determined by quantitative PCR (Fig. 3B). These results indicate that cytoplasmic KpRtgX promotes GDH2 and PEPCK accumulation through post-transcriptional regulation, analogous to the function of cytosolic KpRtg1 [17–19].

During methanol metabolism, synthesis of alcohol oxidase (AOX) and dihydroxyacetone synthase (DHAS) is strongly induced and can be readily detected by SDS–PAGE of lysates of *GS115* cultured in YNBM (Fig. 3C, lane 1). The identity of these bands was established by mass spectrometry in a previous study [34]. In *∆KprtgX*, both AOX and DHAS were downregulated (Fig. 3C, lane 2). Reintroduction of wild-type KpRtgX, but not KpRtgX-NES, restored their expression (Fig. 3C, lanes 3 and 4). Furthermore, *AOX1* mRNA was significantly downregulated in *∆KprtgX* and *KprtgX-NES* (Fig. 3D), and β-galactosidase activity from an *AOX1* promoter-driven *lacZ* reporter was strongly diminished (Fig. 3E). These observations demonstrate that nuclear KpRtgX is required for transcriptional activation of *AOX1* during methanol metabolism, analogous to the role of KpRtg1 in regulating *AOX1* expression [17]. Taken together, these results reveal a dual role for KpRtgX: (i) in the cytoplasm, it post-transcriptionally regulates GDH2 and PEPCK during glutamate metabolism, and (ii) in the nucleus, it activates *AOX1* transcription and promotes methanol metabolism.

### KpRtg1 bHLH domain is essential for its function in glutamate and methanol metabolism

**Figure 4.**
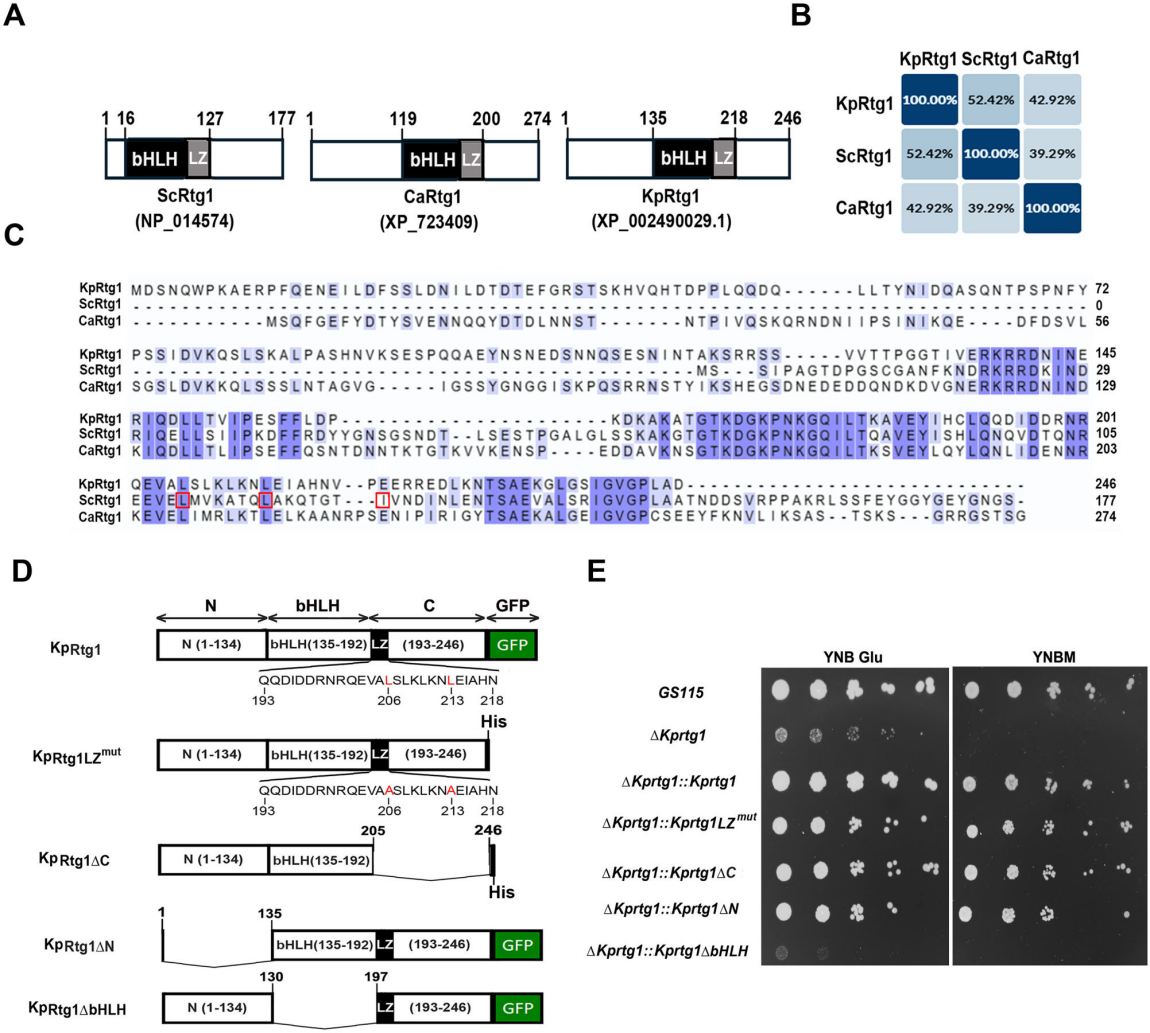
Comparison of Rtg1 of *S. cerevisiae, C. albicans*, and *K. phaffii* and functional characterization of KpRtg1. (**A**) Schematic representation of ScRtg1, CaRtg1, and KpRtg1. NCBI accession numbers are shown in parentheses. bHLH and LZ domains are indicated. Numbers indicate the positions of amino acids. (**B**) Percent identity matrix comparing amino acid sequence identity among ScRtg1, CaRtg1, and KpRtg1. (**C**) Multiple sequence alignment of ScRtg1, CaRtg1, and KpRtg1. Amino acids identical in at least two proteins are shown in violet colour. Numbers indicate amino acid positions. L110, L117, and I124 that contribute to leucine zipper formation in ScRtg1 are boxed. (**D**) Schematic representation of KpRtg1-GFP. Leucine residues at positions 203 and 213 are shown in red. Key functional domains are indicated. His-tagged and GFP-tagged KpRtg1 mutants are shown. In KpRtg1LZ^mut^, L203 and L213 are mutated to alanine. Deletion strains are as indicated in the schematic. (**E**) Analysis of growth of different *K. phaffii* strains by spot assay on YNB Glu and YNBM agar plates.

The remarkable similarity between the phenotypes of *∆KprtgX* (this study) and *∆Kprtg1* [17–19] as well as the regulation of the same target genes by KpRtg1 and KpRtgX during glutamate and methanol metabolism led us to investigate both these proteins in detail. Amino acid sequence analysis revealed that KpRtg1 shares 52.42% and 42.92% identity with ScRtg1 and CaRtg1, respectively, underscoring notable divergence among orthologs (Fig. 4A and B). In ScRtg1, leucine residues at positions 110 and 117 and isoleucine at 124 contribute to leucine zipper formation and dimerization with ScRtg3. Sequence alignments confirmed conservation of L110 and L117 in KpRtg1 and CaRtg1, but not I124 (Fig. 4C). The two conserved leucines in KpRtg1 are at positions 206 and 213 amino acids (Fig. 4C). A series of KpRtg1 mutants were constructed as His-tagged or GFP fusion proteins as indicated in Fig. 4D. These constructs were introduced into the *ΔKprtg1* background, and their ability to restore growth was assessed by spot assays performed in glutamate- and methanol-containing medium. Mutation of leucine residues at 206 and 213 to alanine (KpRtg1LZ^mut^), deletion of C-terminal region containing the LZ (KpRtg1ΔC), and deletion of N-terminal 134 amino acids (KpRtg1ΔN) had no noticeable impact on growth (Fig. 4E). In contrast, removal of the bHLH domain (KpRtg1∆bHLH) completely abolished growth (Fig. 4E). These results indicate that the bHLH domain is essential for KpRtg1 function during glutamate and methanol metabolism, whereas the N- and C-terminal regions as well as the LZ are dispensable.

### KpRtgX and KpRtg1 interact when expressed in *K. phaffii* but not in *E. coli*

**Figure 5.**
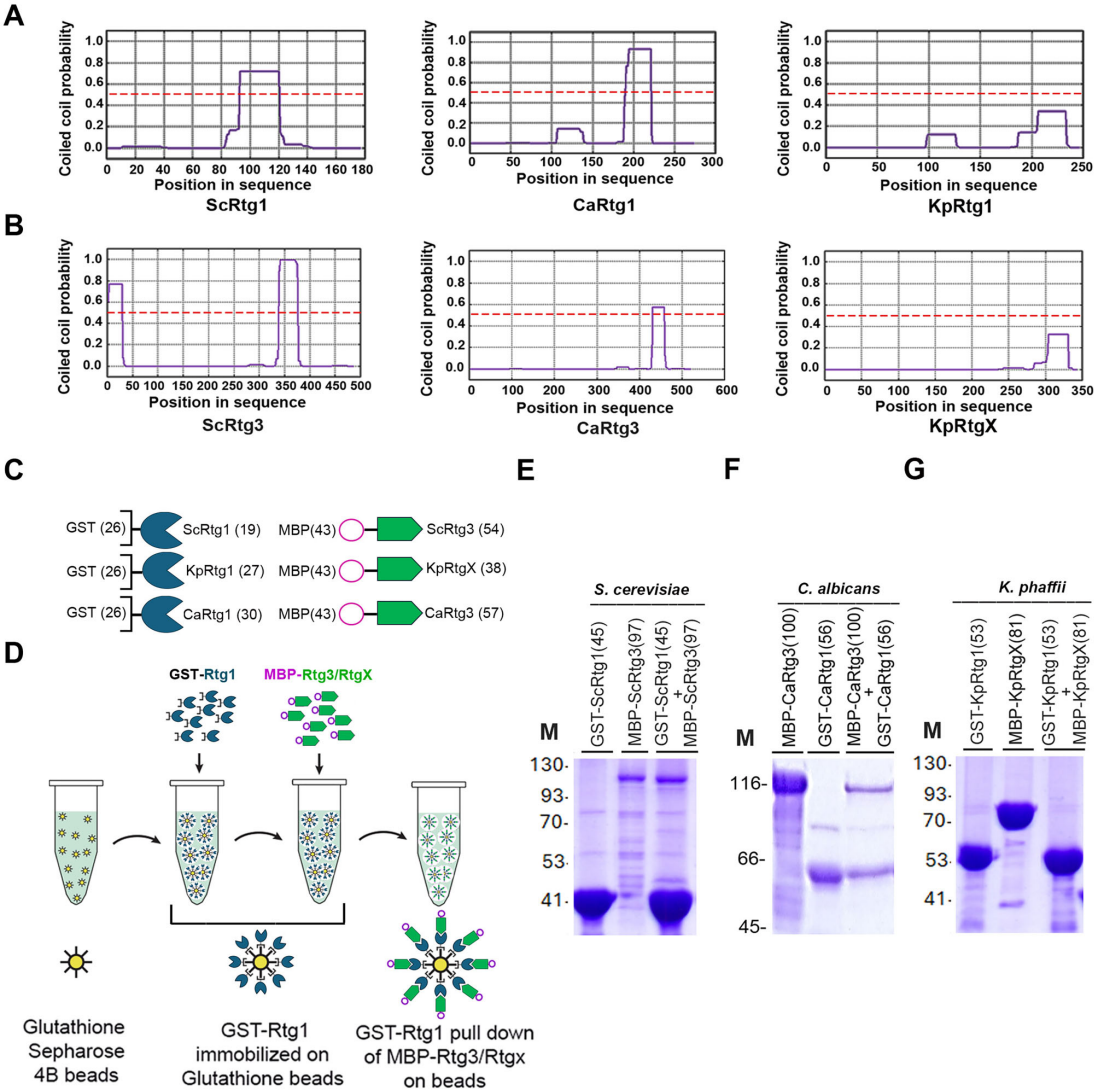
Prediction of coiled-coil domains in Rtg1 and Rtg3/RtgX and analysis of recombinant Rtg1–Rtg3 interactions *in vitro*. (**A, B**) Prediction of coiled coil domains in ScRtg1, CaRg1, KpRtg1, ScRtg3, CaRtg3, and KpRtgX using the prediction tool MARCOIL (https://toolkit.tuebingen.mpg.de/tools/marcoil). The red dotted line at 0.5 indicates a high probability of coiled-coil domain formation. (**C**) Schematic diagram of GST-Rtg1 and MBP-Rtg3 fusion proteins expressed in *E. coli*. Molecular weight of proteins is shown in parentheses. (**D**) Schematic diagram of GST pull-down assay. GST-Rtg1 present in *E. coli* cell lysates was immobilized onto glutathione Sepharose-4B beads. *Escherichia coli* cell lysates containing MBP-Rtg3/RtgX were added and after washing, beads containing Rtg1–Rtg3/RtgX complexes were analysed on SDS–PAGE. (**E, F, G**) Visualization of Rtg1 and Rtg3/RtgX of *S. cerevisiae, C. albicans*, and *K. phaffii*, as indicated by Coomassie Brilliant Blue staining after SDS–PAGE. (M), protein molecular weight markers (kDa). Molecular weight of proteins is shown in parentheses.

Computational prediction identified coiled-coil domains in ScRtg1, CaRtg1, ScRtg3, and CaRtg3, but these domains were absent in KpRtg1 and KpRtgX (Fig. 5A and B). To directly assess heterodimer formation, Rtg1 orthologs were expressed as GST-fusion proteins, and Rtg3 orthologs as MBP-fusion proteins in *Escherichia coli* (Fig. 5C). In GST pull-down assays (Fig. 5D), MBP-ScRtg3 robustly interacted with GST-ScRtg1 (Fig. 5E), and MBP-CaRtg3 with GST-CaRtg1 (Fig. 5F), respectively. Conversely, no interaction was detected between MBP–KpRtgX and GST–KpRtg1 (Fig. 5G). These results indicate that recombinant KpRtgX and KpRtg1 expressed in *E. coli* do not interact *in vitro*, unlike their *S. cerevisiae* and *C. albicans* homologs, reflecting differences in sequence and structural domains essential for dimerization.

**Figure 6.**
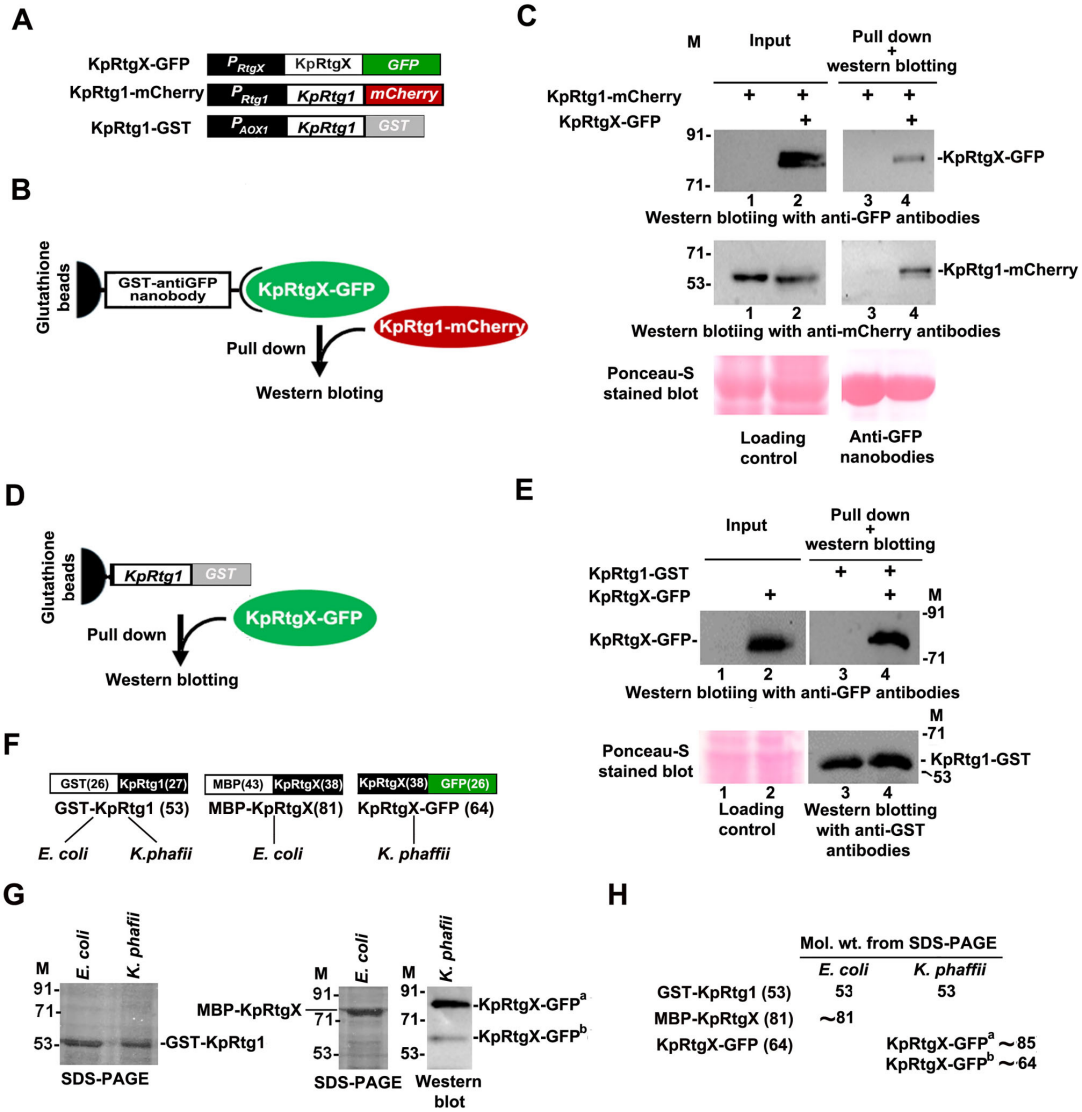
Analysis of interaction between KpRtg1 and KpRtgX by pull-down assays. (**A**) Schematic representation of KpRtgX–GFP, KpRtg1–mCherry, and KpRtg1–GST. (**B**) Schematic representation of pull-down assay. (**C**) Results of western blot analysis after pull-down as indicated. See text for details. (M), protein molecular weight in kDa. (**D**) Schematic representation of pull-down assay. (**E**) Results of western blot analysis after pull-down as indicated. See text for details. (M), protein molecular weight in kDa. (**F**) Schematic representation of GST–KpRtg1, MBP–KpRtgX, and KpRtgX–GFP expressed in *E. coli* and/or *K. phaffii*. Predicted molecular weight is indicated in parentheses. (**G**) Determination of molecular weight of different proteins by SDS–PAGE or western blotting as indicated. (M), protein molecular weight markers in kDa. (**H**) Summary of results obtained in Fig. 6G.

The inability of recombinant KpRtgX and KpRtg1 expressed in *E. coli* to interact with each other *in vitro* and the remarkable similarity in the regulatory features of KpRtg1 and KpRtgX prompted us to investigate the possibility of functional interaction between them during glutamate as well as methanol metabolism in *K. phaffii*. To examine whether KpRtgX and KpRtg1 interact in *K. phaffii*, we expressed KpRtgX as a GFP-fusion (KpRtgX–GFP) and KpRtg1 as an mCherry-fusion (KpRtg1–mCherry) from their native promoters (Fig. 6A). KpRtg1 was also expressed as a GST-tagged protein from methanol-inducible *P_AOX1 _*(Fig. 6A). Lysates were prepared from cells cultured in YNB Glu, and pull-down assays were carried out using anti-GFP nanobodies as bait, as illustrated in Fig. 6B. Lysates containing both KpRtgX–GFP and KpRtg1–mCherry, as well as lysates containing only KpRtg1–mCherry, were used in parallel. KpRtg1–mCherry was successfully pulled down only in the presence of KpRtgX–GFP, whereas no signal was detected from lysates lacking KpRtgX–GFP (Fig. 6C). To investigate KpRtg1 and KpRtgX interaction during methanol metabolism, a reverse pull-down assay was performed using KpRtg1–GST as bait (Fig. 6D). GST-tagged KpRtg1, immobilized on glutathione beads, was incubated with lysate containing KpRtgX–GFP, prepared from *K. phaffii* cells cultured in YNBM. Western blot analysis confirmed that KpRtg1–GST was able to pull down KpRtgX–GFP (Fig. 6E). Thus, KpRtg1 and KpRtgX interact during glutamate as well as methanol metabolism.

To explore why complex formation was observed in *K. phaffii* but not in *E. coli*, we compared the apparent molecular masses of the proteins in both systems. When expressed as GST, MBP, or GFP fusions (Fig. 6F), GST–KpRtg1 migrated as expected (53 kDa) in both hosts (Fig. 6G). MBP-RtgX also migrated as an ∼81 kDa protein as predicted (Fig. 6G). Strikingly, KpRtgX–GFP expressed in *K. phaffii* migrated as two distinct species: a predominant ∼85 kDa band (KpRtgX–GFP^a^) and a minor ∼64 kDa band (KpRtgX–GFP^b^) against the predicted molecular mass of ∼64 kDa (Fig. 6G). The ∼20 kDa upshift of KpRtgX–GFP^a^ suggests the occurrence of post-translational modification(s) in *K. phaffii*. Such modification(s) may promote or stabilize heterodimeric complex formation between KpRtgX and KpRtg1, thereby explaining the host-dependent interaction. These results are summarized in Fig. 6H.

### KpRtgX interacts with KpRtg1 through arginine residues within its bHLH domain and KpRtg1 is essential for the stability of KpRtgX

**Figure 7.**
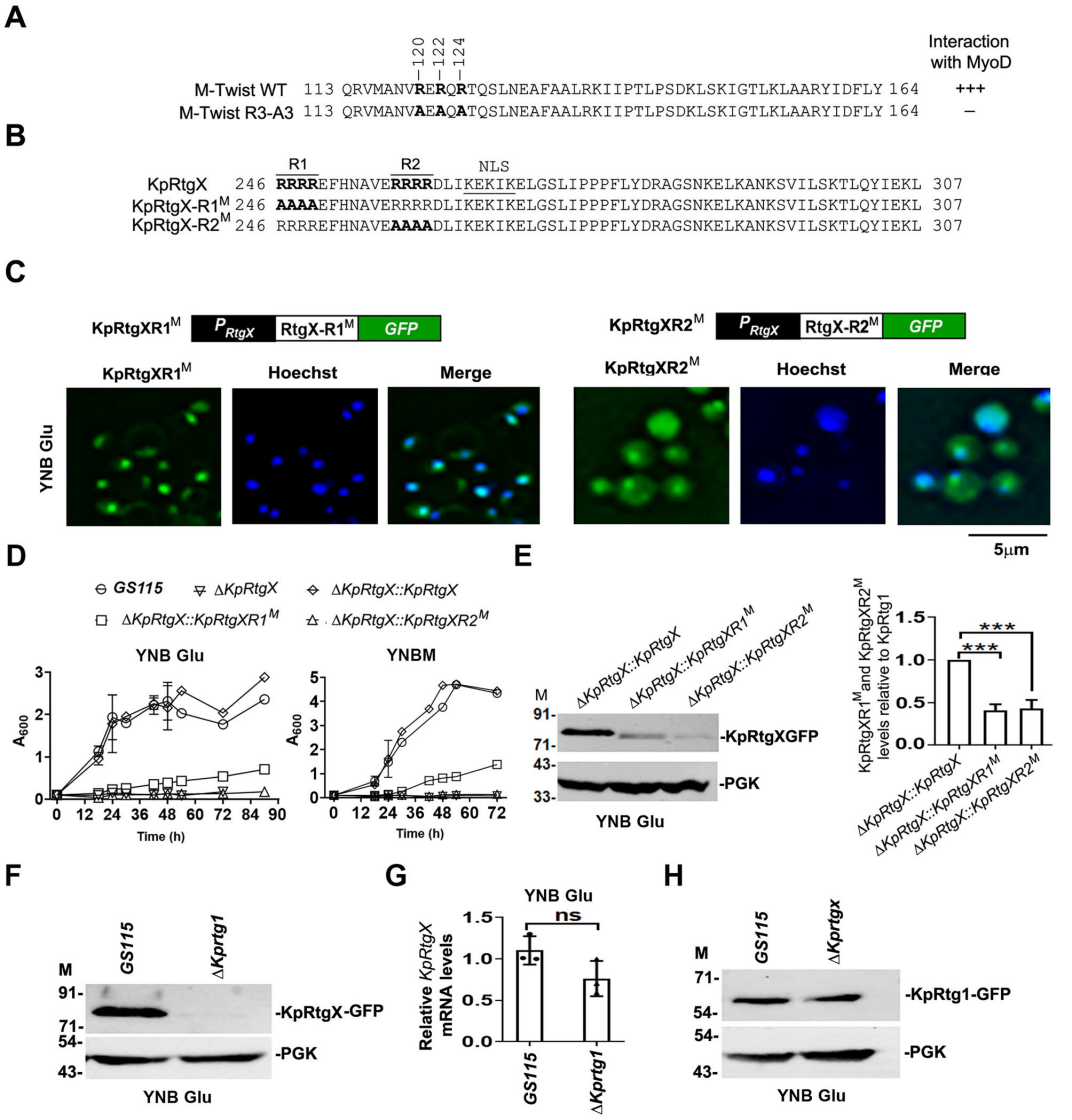
Analysis of interaction between KpRtgX and KpRtg1 expressed in *K. phaffii*. (**A**) Amino acid sequence of bHLH region of mouse Twist (mTwist). Arginine residues involved in MyoD dimerization are shown and has been described [30]. (**B**) Amino acid sequence of bHLH region of KpRgX, KpRtgX-R1^M^, and KpRtgXR2^M^. Arginine-rich clusters spanning residues 246–249 (R1) and 257–260 (R2) and NLS are shown. (**C**) Subcellular localization of KpRtgXR1^M^ and KpRtgXR2^M^ as determined by fluorescence microscopy. (**D**) Analysis of growth of different *K. phaffii* strains cultured in YNB Glu and YNBM for 72h as indicated. Error bars indicate mean ± S.D. *n *= 3. (**E**) Western blot analysis of KpRtgX, KpRtgX-R1^M^, and KpRtgXR2^M^. PGK served as a loading control. Quantitation of bands in panel (E) is shown in the right panel. Error bars indicate mean ± S.D. *n* = 3. (**F**). Western blot analysis of KpRtgX–GFP levels in *GS115* and *ΔKprtg1*. PGK served as a loading control. (M), protein molecular weight markers in kDa. (**G**) Analysis of KpRtgX mRNA levels by qPCR in *GS115* and *ΔKprtgX*. Error bars indicate mean ± S.D. *n *= 3. (**H**) Western blot analysis of KpRtg1-GFP levels in *ΔKprtgX*. PGK served as a loading control. (M), protein molecular weight markers in kDa. Anti-GFP and anti-PGK antibodies were used in western blots.

Given that the bHLH domain, rather than a leucine zipper, is essential for KpRtg1 function, we hypothesized that this domain may mediate its interaction with KpRtgX. KpRtgX also harbours a bHLH domain and lacks a canonical leucine zipper, suggesting that dimerization may proceed through the bHLH domains of both the proteins. In the bHLH proteins m-Twist and MyoD, the amphipathic helices of the HLH region function as dimerization modules, with basic amino acid residues in the juxtaposed regions contributing to partner recognition [35]. Arginine residues at positions 120, 122, and 124 are required for the interaction of m-Twist with MyoD, and mutation of these residues to alanine disrupts heterodimer formation [35] (Fig. 7A). Sequence analysis revealed that KpRtgX contains two arginine-rich clusters within the bHLH domain, spanning residues 246–249 (R1) and 257–260 (R2) (Fig. 7B). To determine whether these arginines contribute to the interaction with KpRtg1, we generated two mutant derivatives of KpRtgX in which the arginines in R1 and R2 clusters were substituted with alanines (designated KpRtgX-R1^M^ and KpRtgX-R2^M^, respectively) (Fig. 7B). Both mutants when expressed as GFP fusion proteins in the *ΔKprtgX* strain localized to the nucleus (Fig. 7C), indicating that mutation of these residues did not impair nuclear targeting. Neither of the mutants was able to rescue the growth defect of *ΔKprtgX* in glutamate- and methanol-containing media (Fig. 7D), suggesting a critical role for R1 and R2 in KpRtgX function during methanol and glutamate metabolism. Immunoblot analysis revealed that the steady-state levels of both KpRtgX-R1^M^ and KpRtgX-R2^M^ were reduced by ~50% relative to KpRtgX (Fig. 7E). These findings suggest that the arginine clusters within the bHLH domain are likely to contribute to KpRtgX–KpRtg1 interaction *in vivo*, and disruption of this interaction compromises KpRtgX stability. Consistent with this notion, KpRtgX protein but not mRNA levels were markedly reduced in the *ΔKprtg1* mutant (Fig. 7F and G), whereas KpRtg1 abundance remained unaffected (Fig. 7H) in the *ΔKprtgX* strain. Together, these data demonstrate that KpRtg1 is required for maintaining KpRtgX stability *in vivo.*

### KpRtg1 regulates the stability of *K. phaffii* GDH2 during glutamate metabolism

**Figure 8.**
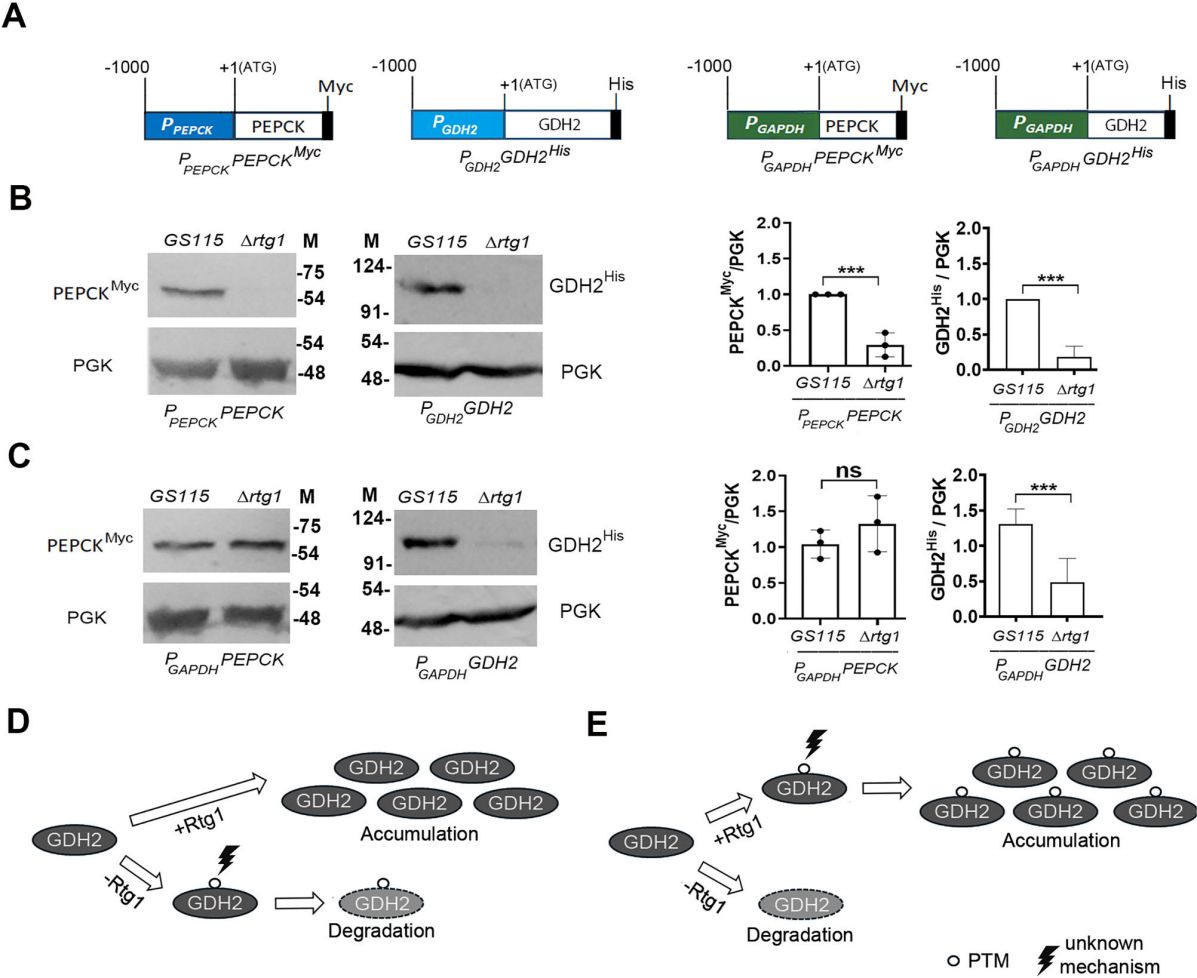
Differential regulation of GDH2 and PEPCK by *K. phaffii* Rtg1. (**A**) Schematic representation of PEPCK and GDH2 expression constructs. (**B, C**) Western blot analysis of PEPCK^Myc^ and GDH2^His^ using anti-Myc and anti-His antibodies, respectively. PGK served as a loading control. Cell lysates were prepared after 6 h of growth in YNB-Glu medium. (M), protein molecular weight markers (kDa). Quantification of western blot signals is shown in the right panels. Statistical significance is indicated as follows: *P < .05; **P < .005; ***P < .0005; ns, not significant. Error bars represent the mean ± S.D. from three independent biological replicates. (**D**) A model for post-transcriptional regulation of GDH2 by KpRtg1. KpRtg1 is necessary for GDH2 stability, and in its absence, GDH2 undergoes post-translational modification and is targeted for degradation. (**E**) An alternate model suggests that GDH2 undergoes KpRtg1-dependent post-translational modification, which confers stability, and in the absence of KpRtg1, the unmodified GDH2 is degraded. PTM, post-translational modification.

To investigate the mechanism of post-transcriptional regulation of PEPCK and GDH2 by KpRtg1, we expressed PEPCK and GDH2 tagged with Myc and His epitopes (PEPCK^Myc^ and GDH2^His^, respectively) either from their native promoters (*P_PEPCK_PEPCK^Myc^, P_GDH2_GDH2^His^*) or from the constitutive *GAPDH* promoter (*P_GAPDH_PEPCK^Myc^, P_GAPDH_GDH2^His^*) (Fig. 8A). The constructs were introduced into wild-type (*GS115)* and *ΔKprtg1* strains, which were initially grown in YPD medium and then shifted to YNB-Glu. After 6 h, protein extracts were analysed by western blotting using anti-Myc and anti-His antibodies, respectively. When expressed from their native promoters, both PEPCK^Myc^ and GDH2^His^ were downregulated in the *ΔKprtg1* background (Fig. 8B). Interestingly, GDH2^His^ but not PEPCK^Myc^ was downregulated in *ΔKprtg1* even when driven by the *GAPDH* promoter (Fig. 8C). This suggests that Rtg1 regulates PEPCK and GDH2 through distinct mechanisms. The promoter-independent downregulation of GDH2^His^ in *ΔKprtg1* implies that KpRtg1 may regulate GDH2 protein levels by influencing protein stability. One possibility is that KpRtg1 prevents GDH2 degradation, and in its absence, the protein undergoes post-translational modifications leading to degradation (Fig. 8D). Alternatively, KpRtg1 may facilitate a stabilizing modification, and in *ΔKprtg1*, the unmodified protein becomes unstable and is targeted for degradation (Fig. 8E). Further studies are required to distinguish between these possibilities and to define the precise role of KpRtg1 in regulating GDH2 protein levels.

### Translational regulation of *K. phaffii PEPCK* mRNA by KpRtg1

**Figure 9.**
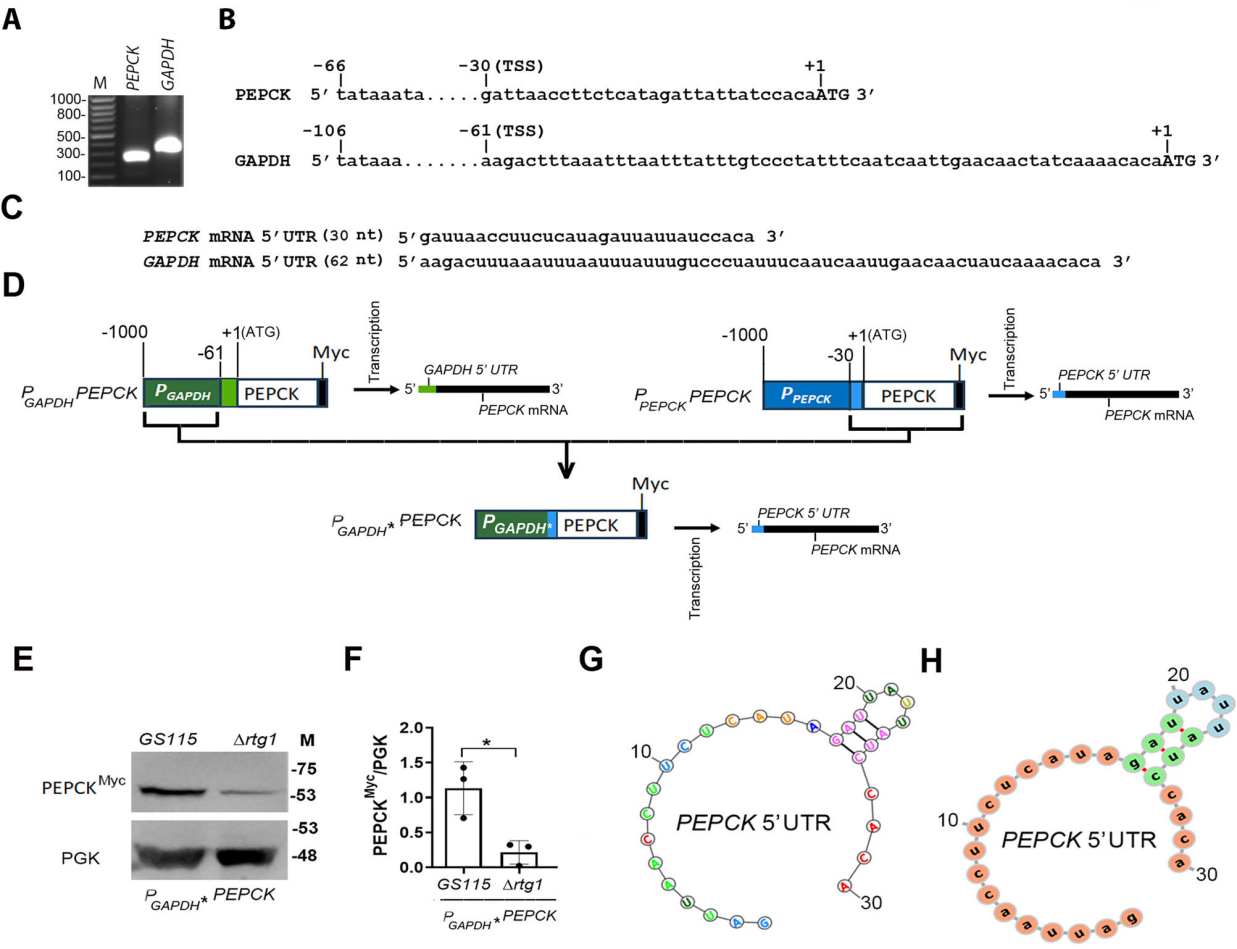
KpRtg1 regulates PEPCK expression through the 5′ UTR of *PEPCK* mRNA. (**A**) Agarose gel electrophoresis of final 5′ RLM-RACE products to determine TSS of *K. phaffii PEPCK* and *GAPDH*. (M), DNA molecular weight markers (bp). (**B**) Nucleotide sequence of 5′ RACE products as determined by DNA sequencing using antisense primers specific to the target genes. TSSs are indicated. (**C**) Nucleotide sequence of 5′ UTRs of *K. phaffii PEPCK* and *GAPDH* mRNAs deduced from panel (B). (**D**) Schematic representation of the strategy for the construction of P_GAPDH_*PEPCK in which the 5′ UTR of *K. phaffii P_GAPDH_* was replaced with that of *P_PEPCK_*. The *PEPCK* mRNA transcribed from P_GAPDH_*PEPCK consists of *PEPCK* 5′ UTR. (**E**) Western blot analysis of PEPCK^Myc^ using anti-Myc antibodies. PGK served as a loading control. Cell lysates of *GS115* and *ΔK prtg1* expressing PEPCK^Myc^ from P_GAPDH_* were prepared after 6 h of growth in YNB-Glu medium. (M), protein molecular weight markers (kDa). (**F**) Quantification of western blot signals is shown in the right panels. Statistical significance is indicated as follows: *P < .05; **P < .005; ***P < .0005; ns, not significant. Error bars represent the mean ± S.D. from three independent biological replicates. (**G, H**) Secondary structure prediction within the *PEPCK* 5′ UTR by two RNA secondary structure prediction web servers (https://rna.urmc.rochester.edu/RNAstructureWeb/Servers/Predict1/Predict1.html; http://rna.tbi.univie.ac.at/forna/).

The region upstream of initiation codon of a eukaryotic mRNA is referred to as the 5′ untranslated region (5′ UTR), and it corresponds to the region between the transcription start site (TSS) and one nucleotide before the start codon of the corresponding gene. 5′ UTRs often play an important role in the regulation of translation of mRNAs. 5′ UTRs harbour several regulatory elements such as upstream ORFs, internal ribosome entry sites, microRNA binding sites, binding sites for structural components involved in the regulation of mRNA stability, pre-mRNA splicing, and hairpin/stem-loop structures that serve as binding sites for RNA-binding proteins regulating translation initiation [36, 37]. Since the TSS of *K. phaffii PEPCK* is not known, we first mapped the TSS of *PEPCK* together with the TSS of *K. phaffii GAPDH* using RNA ligase-mediated rapid amplification of cDNA ends using the 5′ RLM RACE kit (Ambion, USA) as per the manufacturer’s instructions. *Komagataella phaffii PEPCK* and *GAPDH* 5′ products obtained from RLM-RACE were resolved on an agarose gel (Fig. 9A), and the TSSs were determined by DNA sequencing using antisense primers homologous to the 3′ end of the PCR products. TSSs of *PEPCK* and *GAPDH* were mapped at −30 and −61 bp, respectively (Fig. 9B). The 5′ UTRs of *PEPCK* and *GAPDH* mRNAs were deduced from the DNA sequence (Fig. 9C). Since PEPCK is downregulated in *∆Kprtg1* only when transcribed from *P_PEPCK_* (Fig. 8B) but not *P_GAPDH_* (Fig. 8C), we generated P_GAPDH_*PEPCK^Myc^ in which −1000 to −61 bp of the *GAPDH* promoter was fused to −30 bp of *PEPCK* 5′ UTR and *PEPCK^Myc^* coding region (Fig. 9D). Thus, *PEPCK* mRNA generated from P_GAPDH_*PEPCK^Myc^ will contain *PEPCK* 5′ UTR rather than *GAPDH* 5′ UTR (Fig. 9D). This construct was introduced into both *GS115* and *ΔKprtg1* strains, and protein lysates from cells grown in YNB Glu for 6 h were analysed by western blot using anti-Myc antibodies. PEPCK expression in *ΔKprtg1* was significantly lower in P_GAPDH_* than that from *P_GAPDH_* in the *ΔKprtg1* strain (compare Fig. 9E and F with Fig. 8C). This indicates that synthesis of PEPCK from *GAPDH* promoter becomes KpRtg1-dependent when its 5′ UTR is substituted with that of *PEPCK* promoter. *In silico* analysis for the presence of secondary structures in the *PEPCK* 5′ UTR using two RNA secondary structure prediction tools (https://rna.urmc.rochester.edu/RNAstructureWeb/Servers/Predict1/Predict1.html; http://rna.tbi.univie.ac.at/forna/) resulted in the identification of a putative short stem-loop structure within this region (Fig. 9G and H). The involvement of these stem-loop structures in KpRtg1-dependent translation of *PEPCK* mRNA remains to be elucidated.

## Discussion

Our study identifies the previously uncharacterized bHLH protein KpRtgX as the heterodimeric partner of KpRtg1. KpRtg1–KpRtgX complex coordinates both transcriptional and post-transcriptional regulation of key metabolic enzymes in *K. phaffii* during glutamate and methanol metabolism. While *K. phaffii* has been widely studied for its metabolic versatility and industrial potential, the molecular mechanisms that enable its adaptive use of diverse carbon sources have remained largely undefined. Here, we uncover a unique regulatory pathway centered around these two bHLH proteins, with functions distinct from their homologs in *S. cerevisiae and C. albicans*. Although KpRtgX was originally annotated as a putative Rtg3 homolog, our findings reveal that it functions in a fundamentally different manner than its *S. cerevisiae* and *C. albicans* counterparts. Remarkably, we show that the bHLH domain of KpRtg1 alone is sufficient for regulatory function, while its leucine zipper, essential for dimer formation in other yeasts, is dispensable in *K. phaffii*. Conversely, KpRtgX function and stability critically depend on conserved arginine residues within its bHLH domain, which facilitate interaction with KpRtg1. One particularly striking feature of KpRtgX is its localization-specific function. During methanol metabolism, nuclear KpRtgX acts as a transcriptional activator of *AOX1*. During glutamate metabolism, cytosolic KpRtgX is sufficient for post-transcriptional regulation of *GDH2* and *PEPCK*. This compartment-specific regulatory function mirrors mechanisms observed in higher eukaryotes, where transcription factors often shuttle between the nucleus and cytoplasm to perform distinct roles. Moreover, the inability of KpRtgX to interact with KpRtg1 when expressed in *E. coli*, alongside evidence of possible post-translational modification in *K. phaffii*, highlights the importance of host-specific modification in enabling proper protein function and complex formation.

**Figure 10.**
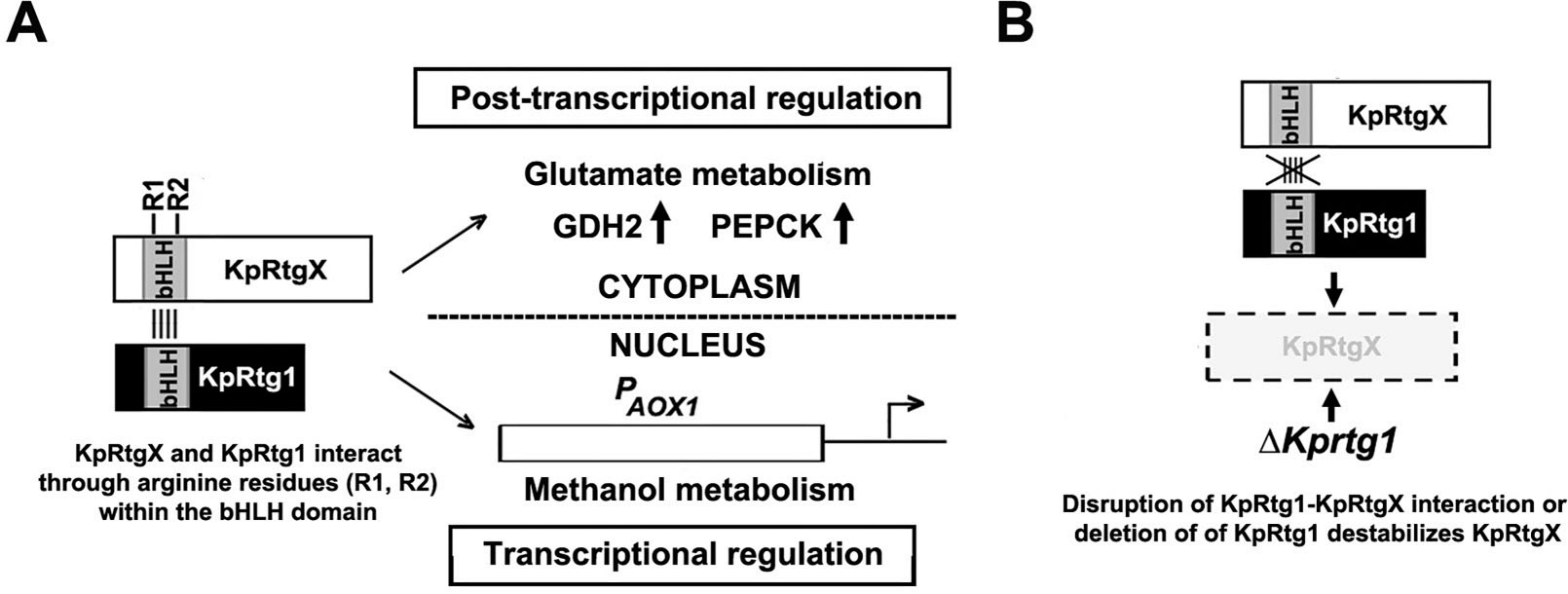
Schematic representation of dual regulatory functions of the KpRtg1–KpRtgX complex in *K. phaffii*. (**A**) Schematic representation of the dual roles of the KpRtg1–KpRtgX complex in *K. phaffii* under different metabolic conditions. KpRtgX contains two arginine-rich clusters (R1: residues 246–249; R2: residues 257–260) within its bHLH domain, which are essential for its interaction with the bHLH domain of KpRtg1. During methanol metabolism, the KpRtg1–KpRtgX complex localized in the nucleus acts as a transcriptional activator of *AOX1*. During glutamate metabolism, the complex localized in the cytoplasm serves as a post-transcriptional regulator, promoting the accumulation of GDH2 and PEPCK proteins. (**B**) Disruption of the KpRtg1–KpRtgX interaction, either by alanine substitution of arginines in the R1 and R2 clusters or by deletion of KpRTG1 (*ΔKprtg1*), leads to instability of KpRtgX. These observations highlight the essential roles of both the physical interaction between KpRtg1 and KpRtgX and the correct subcellular localization of the complex for its dual regulatory functions in methanol and glutamate metabolic pathways.

Transcription factors regulate gene expression by binding to specific promoter sequences of the target genes, and Rtg1–Rtg3 is no exception. In *S. cerevisiae*, Rtg1–Rtg3 regulates the transcription of *CIT2* encoding peroxisomal citrate synthase by binding to the R box element (GTCAC sequence) in the promoter [21, 23, 35]. In *C. albicans*, Rtg1–Rtg3 binds to the region from −1270 to −1077 bp in the promoter of *AOX2* encoding alternate oxidase harbouring three critical GTCA sequence motifs, and mutation of the GTCA motifs abolishes Rtg1–Rtg3 binding [28]. Since β-galactosidase expression from *P_AOX_-LacZ* is abrogated in *∆KprtgX*, the KpRtg1–KpRtgX binding site is likely to be present within −1000 bp of *AOX1* promoter, which remains to be identified. While we focused our attention only on the regulation of key enzymes of glutamate and methanol metabolism, the growth defects observed in Δ*Kprtg1* and Δ*KprtgX* strains across various other carbon sources including glutamate, ethanol, and acetate indicate a central role of this complex in the regulation of multiple metabolic pathways. Interestingly, deletion of *KpRtg1* also leads to severe downregulation of *KpRtgX*, suggesting a hierarchical relationship, with KpRtg1 likely acting as a master regulator. A novel and particularly compelling aspect of our findings is the post-transcriptional regulation of *PEPCK* and *GDH2 by KpRtg1*. We demonstrate that KpRtg1 modulates *PEPCK* expression via its 5′ UTR, a regulatory mechanism not previously described in yeast. Additionally, KpRtg1 regulates GDH2 by a distinct mechanism, by influencing protein stability. Since KpRtgX is downregulated in ∆Kprtg1, similar results can be expected in ∆KprtgX as well. The mechanism awaits further investigation. Key findings of this study are summarized in Fig. 10.

This study reveals an unexpected repurposing of conserved eukaryotic signaling pathways in *K. phaffii*, wherein bHLH transcription factors have evolved to integrate transcriptional and post-transcriptional control in response to nutrient availability. This mode of regulation reflects a specialized adaptation in methylotrophic yeasts, enabling efficient switching between preferred and non-preferred carbon sources. More broadly, our work underscores the emerging importance of RNA-level regulation by transcription factors in fungi and establishes a foundation for deeper mechanistic studies. Determining how factors such as KpRtg1 and KpRtgX are modulated through subcellular localization, post-translational modification, or protein–protein interactions will be essential for leveraging these systems in synthetic biology and metabolic engineering. These insights open opportunities to design *K. phaffii* strains with precisely tuned metabolic programs for industrial bioprocessing. Finally, our findings show that carbon source-dependent divergence of bHLH regulatory functions represents a previously unrecognized principle of nutrient-driven pathway rewiring, with broad implications for the evolution of metabolic control networks.

## Supplementary Material

gkaf1475_Supplemental_File

## Data Availability

The data underlying this article are available in the article and its online supplementary material.
