# Supplementary material for: Dual transcriptional and post-transcriptional regulation by the bHLH proteins Rtg1 and RtgX in Komagataella phaffii
Source: Nucleic Acids Res. 2026 Jan 8;54(1):gkaf1475. doi: 10.1093/nar/gkaf1475 (PMC12781876; doi:10.1093/nar/gkaf1475)
Supplement: gkaf1475_Supplemental_File [file gkaf1475_supplemental_file.pdf]

Supplementary material

Dual transcriptional and post-transcriptional regulation by the bHLH proteins Rtg1 and RtgX in *Komagataella phaffii*

Neetu Rajak, Richa Shah, Yash Sharma and Pundi Rangarajan*

Department of Biochemistry, Indian Institute of Science, Bangalore 560012, INDIA

Table S1: List of qPCR and RACE primers*.

Primer	Primer sequence (5' - 3')
18S qFP	AATGAGGATTGACAGGATGA
18S qRP	AGGTCTCGTTCGTTATCG
GDH2 qFP	ACGAAGAACGCATTGATG
GDH2 qRP	CGAAGTAGCAACGAACATTA
PEPCK qFP	ATGATGAGCACTGTTGGT
PEPCK qRP	CCGAACTTGATAGCATTGAA
AOX1 qFP	AACTTGTCTGCTGGTTCTT
AOX1 qRP	CCTTGTCATCCTCCTCAT
Rtg1 qFP	CATTGATGTTAAGCAGTCTCT
Rtg1 qRP	GATTCGGATTGATTGTTGGA
RtgX qFP	CTCCGTATGACTTAGGTTCT
RtgX qRP	GTATGAATTATCCAGGCTATTGTA
Outer primer FP	GCTGATGGCGATGAATGAACACTG
Inner primer FP	CGCGGATCCGAACACTGCGTTTGCTGGCTTTGATG
RP_PEPCK_RLM_1	TTT GGA GAT CTT CCG GTC TTC TTA CC
RP_PEPCK_RLM_2	ATA GTC GAT GGC TCT GGC CT
RP_GAP_RLM_1	TGTTGACAGGGTCTCTCTCTTGGAAAACGG
RP_GAP_RLM_2	CTTCTTGGCACCGGCGTCGATG

*See materials and methods for details.

Table S2: List of PCR primers used for the generation of various *K. phaffii* strains[*].

Primer	Primer Sequence (5′ - 3′)
A1	GTCGAATTCGAGCTCGGTACGGTTTCAAATCTGAGTTGCTT
A2	ATT ATG AGC AAT CTC TGC GTT TTT GAG TTT CAA GGA CGC AGC<br>GAC CTC TTG ACG
A3	CGT CAA GAG GTC GCT <u>GCG</u> TCC TTG AAA CTC AAA AAC <u>GCA</u> GAG<br>ATT GCT CAT AAT (mutated bases are underlined)
A4	GAGACTAGTGGATCCCCGGTCAATGGTGGTGGTGATGATGATCTGCT<br>AGTGGTCCAAC
B1	CGA GAC TAG TGG ATC CCC GGT CAA TGG TGG TGG TGA TGA TGA<br>GCG ACC TCT TGA CGG TTT
C1	CGGCCGTCTCGGATCGGTACGGTTTCAAATCTGAGTTGCTTTGGG
C2	TGTCTCTGCGCTTGCGCTCAACCATGAAGGTAGTCTTTCGTAGATGGA<br>GT
C3	ACT CCA TCT ACG AAA GAC TAC CTT C AT GGT<br>TGAGCG CAA GCG CAG AGA
C4	AAAGCTGGCGGCCGCCGCGGCTCGAGGTGGTGGTGGCTAGCTTTGTA<br>TAGTTCAT
D1	CCG<u>CTCGAG</u>GATTTCTGCGATTGGAGCG (XhoI site is underlined)
D2	GACAAAGCGACCTCTTGACGGTTTCTCCC<br>ACCTGGAGTTGTCACCACTGAG
D3	CTCAGTGGTGACAACTCCAGGTGGGAGAAACCGTCAAGAGGTCGCTT<br>TGTC
D4	CCC<u>AAGCTT</u>CTAGTGGTGGTGGCTAGCTTTG (HindIII site is underlined)
E1	GAGTGGAGTATCTGTTCCTCCATG
E2	CTTGATTAGGGGGAAGAAGCTTC
E3	CGTCTTCAAAATTCTTACCATTCTAAGTGCGGATCCCCCACACACCAT<br>AGC
E4	AGACCCTTGGACACATAAAAGGAGGTGCTCAATGTTGGTCTCCAGCT<br>TG
E5	CAAGCTGGAGACCAACATGTGAGCACCTCCTTTTATGTGTCCAAGGG<br>TCT
E6	GCTATGGTGTGTGGGGGATCCGCACTTAGAATGGTAAGAATTTTGAA<br>GACG
E7	CGCGGATCCATGGCACAAGATGCTCCCTTTG
E8	CTCCTTTACTAGTCAGATCTACCATTTGCCTAGACTTGTCGTTATCAG<br>CAGC
F1	CGG<u>GGTACC</u>GGCGTACTTGGTCTCTGGAGGT (KpnI site is underlined)
F2	CTCCTTTACTAGTCAGATCTACCATGTTCGGAACGTCAAGGAAATTAT<br>TAC
F3	ATGGTAGATCTGACTAGTAAAGGAGGTAATAATTTCCTTGACGTTCC<br>GAAC
F4	ATAAGAAT<u>GCGGCCGC</u>CTAGTGGTGGTGGCTAGCTTTG (NotI site is<br>underlined)
G1	TGGTAGATCTGACTAGTAAAGGAGGTAATAATTTCCTTGACGTTCCG

	AAC
G2	ATAAGAATGCGGCCGCCTATAGCTCCAAAGCAGCCAAAGCGTCAGACAAAGCAGACTCGATGTGGTGGTGGC TAG CTT TG TATAG (NES-coding sequence in the reverse primer is underlined.)
H1	CGGCCGTCTCGGATCGGTACCGTCTCTG GAGGTATAGTGGC
H2	CGGCCGCCGCGGCTCGAGGATCCTTAGAATGGTAAGAAT
H3	TCCTCGAGCCGCGGCGTGAGCAAGGGCGAG
H4	TAGAAAGCTGGCGGCCTTAGCGGCCGCTTACTTGTACAGCTCG
I 1	AAA GCA ACT CAG ATT TGA AAC CGA CTA GTG GAT CCC CGG GTA CC
I 2	CGC CCT TGC TCA CCA TGC TCG AAT CTG CTA GTG GTC CAA CAC CA
J1	TAA TTA TTC GAA ACG AGG AAT TCA TGT CCC CTA TAC TAG GTT ATT
J2	CTT GAA AAT ATA AAT TTT CGG TAC CGT CAA TCT GCT AGT GGT CCA ACA
K1	TTC TTA CCA TTC TAA GGG ATC CAT GGC ACA AGA TGC TCC CTT TGA TCT TTT
K2	GACGTTCCACAGCATTGTGGAATTCTGCGGCTGCTGCCATTTTCTCTTCTCTATTCAGAGCA (Mutated bases are underlined)
K3	TGCTCTGAATAGAGAAGAGAAAATGGCAGCAGCCGCAGAATTCCACAATGCTGTGGAACGTC (Mutated bases are underlined)
K4	TTG CTC ACG CCG CGG CTC GAG TTC GGA ACG TCA AGG AAA TTA TTA
L 1	CTTTAATTTTTTCCTTGATAAGGTCTGCGGCTGCTGCTTCCACAGCATTGTGGAATTCTCTT (Mutated bases are underlined)
L 2	AAGAGAATTCCACAATGCTGTGGAAGCAGCAGCCGCAGACCTTATCAAGGAAAAAATTAAAG (Mutated bases are underlined)
M1	GTC GAA TTC GAG CTC GGT ACT TTG AAG TAA AAC TTT AAC TT
M2	TGT GGA TAA TAA TCT ATG AGA AGG TTA ATC GGG TCA GGA GAA ACC AAA ATT G
M3	CAA TTT TGG TTT CTC CTG ACC CGA TTA ACC TTC TCA TAG ATT ATT ATC CAC A
M4	CTA GTG GAT CCC CGG TTA CAG GTC TTC TTC AGA GAT CAG TTT CTG TTC CAA CTG AGG GCC GG

*See materials and methods for details.

Table S3. Nucleotide sequence of synthetic genes procured from Twist Bioscience.

**Synthetic gene**	**Nucleotide sequence (5′-3′)**
KpRtgXK1^{M}	cttcaAaattcttaccattctaagggatcCatggcacaagatgctccctttgatcttttcaccgatactaccaat<br>gattcacatgaaagaaaatcttctataagctataatgatgatgattttgataagtttctaaatttcgaacaaattga<br>caacatgagcccgctacaagaagaccctaccgatactcatttgatgagcaaaatgatttcaggaggagaag<br>catttgaagaaccctttgacttgcaatctcagcaaaatctgcaccaggatttagaatcttttagttcttctgcaaa<br>ccagagctatcaaccacaagatcaatatggctttcaaggtagcaattttgacatagatacttccattgcaacct<br>cttcgggaaattacaatgctggtctattgaattcacagtatttctcacctcgtatcaaaccaacggtaggtagac<br>ctgggactgcaatttcccctcttcccggaactctgtcaacacctcaggttacccagtcagcaactggtcacgc<br>acctgtaagtcatggctctagtacaagtgaagctataaaaattgcatcacctccgtatgacttaggttctgatgc<br>ggggacttcgtttggcactccttttggcacttcgtttggggcagagtcttacaatagcctggataattcatacat<br>aaagtccccatcgttcaaaggaagcttgggatcacctgggactgcgcaaggctctctaagctcaaaaagtg<br>ctctgaatagagaagagaaaatgagacggagaagagaattccacaatgctgtggaacgtcggcggcgag<br>accttatc**GCG**gaa**GCA**att**GCA**gagttaggatcattaataccaccgcctttttgtacgatcgggcg<br>ggttcgaacaaggaacttaaagcaaacaagagtgtcattttgagcaagacccttcaatatatcgaaaagctg<br>agtgagattaaagcatctcaagacatccgcctcaagcatcttcgtgaatgcattgacctttatgaaaacatcga<br>cacgggtaataatttccttgacgttccgaactcgagccgcggcgtgagcaagggcgagga
KpRtgXK2^{M}	cttcaAaattcttaccattctaagggatcCatggcacaagatgctccctttgatcttttcaccgatactaccaat<br>gattcacatgaaagaaaatcttctataagctataatgatgatgattttgataagtttctaaatttcgaacaaattga<br>caacatgagcccgctacaagaagaccctaccgatactcatttgatgagcaaaatgatttcaggaggagaag<br>catttgaagaaccctttgacttgcaatctcagcaaaatctgcaccaggatttagaatcttttagttcttctgcaaa<br>ccagagctatcaaccacaagatcaatatggctttcaaggtagcaattttgacatagatacttccattgcaacct<br>cttcgggaaattacaatgctggtctattgaattcacagtatttctcacctcgtatcaaaccaacggtaggtagac<br>ctgggactgcaatttcccctcttcccggaactctgtcaacacctcaggttacccagtcagcaactggtcacgc<br>acctgtaagtcatggctctagtacaagtgaagctataaaaattgcatcacctccgtatgacttaggttctgatgc<br>ggggacttcgtttggcactccttttggcacttcgtttggggcagagtcttacaatagcctggataattcatacat<br>aaagtccccatcgttcaaaggaagcttgggatcacctgggactgcgcaaggctctctaagctcaaaaagtg<br>ctctgaatagagaagagaaaatgagacggagaagagaattccacaatgctgtggaacgtcggcggcgag<br>accttatcaaggaaaaaattaaagagttaggatcattaataccaccgcctttttgtacgatcgggcgggttcg<br>aac**GCG**gaactt**GCA**gcaaac**GCG**agtgtcattttgagcaagacccttcaatatatcgaaaagctg<br>agtgagattaaagcatctcaagacatccgcctcaagcatcttcgtgaatgcattgacctttatgaaaacatcga<br>cacgggtaataatttccttgacgttccgaactcgagccgcggcgtgagcaagggcgagga

Mutated bases are shown in bold, upper case.
